# Modeling pediatric low-grade glioma heterogeneity using human forebrain organoids

**DOI:** 10.1186/s12943-026-02612-x

**Published:** 2026-04-01

**Authors:** Gloria Leva, Lucia Santomaso, Matteo Gianesello, Sara Patrizi, Federica Ress, Federico Cocchini, Celeste Antonacci, Francesca Gianno, Luana Abballe, Chiara Lago, Noemi Pozza, Gabriele Trentini, Marina Cardano, Simone Minasi, Francesca Romana Buttarelli, Manila Antonelli, Davide Pernici, Linda Petrucci, Francesco Antonica, Emma Busarello, Martina Iannuzzi, Alessia Soldano, Toma Tebaldi, Evelina Miele, Elisabetta Ferretti, Luca Tiberi

**Affiliations:** 1 Department CIBIO, University of Trento, Trento, Italy; 2 Onco-Hematology, Cell Therapy, Gene Therapies and Hemopoietic Transplant, Bambino Gesù Children’s Hospital, IRCCS, Rome, Italy; 3 Department of Radiological, Oncological and Anatomo Pathological Sciences, Sapienza University, Rome, Italy and IRCCS Neuromed, Pozzilli, Italy; 4 Department of Neuroscience, SISSA, Trieste, Italy; 5 Department of Internal Medicine, Section of Hematology, Yale Comprehensive Cancer Center, Yale University School of Medicine, New Haven, CT USA; 6 Department of Experimental Medicine, Sapienza University, Rome, Italy

## Abstract

Pediatric low-grade gliomas (pLGGs) are the most common type of brain tumors in children, characterized by their typically slow growth and oncogene-induced senescence. Preclinical models provide the opportunity to investigate the effects of various treatments in a controlled setting before they are tested in human patients; however, reliable models for pLGGs are limited. Here we developed two organoid models for pLGGs, which, after engraftment into mice, exhibited low-grade features. Furthermore, the genome-wide DNA methylation and RNA profiles of the organoids demonstrated closer similarity to low-grade glioma entities compared to high-grade counterparts. Additionally, pLGG organoid-derived cells align with oligodendrocyte-like, astrocyte-like and MAPK signature clusters seen in patient tumors, indicating that the organoids generate a heterogeneous population of cancer cells, where cellular diversity may influence disease progression and treatment response.

**Supplementary Information:**

The online version contains supplementary material available at https://doi.org/10.1186/s12943-026-02612-x.

## Introduction

Pediatric low-grade gliomas (pLGGs) are the most common type of brain tumors in children, accounting for approximately 30% of all pediatric brain tumors [1]. pLGGs encompass a diverse spectrum of glial, neuronal, and mixed glioneuronal tumors, as classified by the World Health Organization (WHO) within its central nervous system (CNS) tumor taxonomy [2]. Despite their typically slow growth, pLGGs can cause significant symptoms that negatively affect the patient’s quality of life. Common symptoms include headaches, nausea, vomiting, seizures, and changes in behavior or cognitive abilities. Treatment options for pediatric low-grade gliomas include surgery, radiation therapy, and chemotherapy. Patients who survive frequently present with long-term sequelae attributable to both the disease itself and the consequences of treatment [3]. The choice of treatment depends on various factors, including the patient’s age, overall health, tumor size and location, and the specific type of glioma. Recent advancements in molecular profiling of pLGGs have enabled clinical trials evaluating molecular therapies targeting the RAS/MAPK and mTOR pathways. Phase I/II studies have shown promising results with agents such as MEK inhibitors (selumetinib, trametinib, and binimetinib), a pan-RAF inhibitor (tovorafenib), first-generation BRAF inhibitors (vemurafenib and dabrafenib), a mTOR inhibitor (everolimus), and a FGFR inhibitor (erdafitinib). These findings have led to the initiation of randomized controlled trials comparing these agents, including selumetinib, trametinib, and tovorafenib, with standard chemotherapy in newly diagnosed, untreated pLGG patients [4–6].

Preclinical models enable researchers to investigate the effects of candidate treatments in a controlled setting, allowing for in-depth analysis of their mechanisms of action and possible side effects before clinical testing in humans. Additionally, preclinical models serve as platforms for developing and testing novel drugs or treatment combinations, which could enhance patient outcomes for those with pLGG.

A major challenge in generating viable patient-derived cell lines for pLGG is the limited availability of tumor tissue, as these tumors often arise in surgically inaccessible regions and yield only small biopsy samples insufficient for cell line establishment. Additionally, the oncogene-induced senescence (OIS) phenomenon poses another major obstacle in establishing viable cell lines for pLGGs [7, 8]. OIS is characterized by cycle arrest, which is accompanied by the induction of p16^INK4a^ and senescence-associated β-galactosidase (sGAL) activity, commonly used senescence markers [7, 8]. Efforts to develop patient-derived pediatric LGG xenograft (PDX) models have encountered several challenges. Many of these models do not completely recapitulate the molecular and cellular heterogeneity of patients’ tumors, as they have acquired additional genetic alterations [9]. We and others have recently attempted to generate patient-derived tumoroids from LGG tumor specimens, but these tumoroids have shown more difficulties adapting to the in vitro culture conditions compared to those derived from ependymomas and medulloblastomas samples, failing in generating tumors *in vivo* [10, 11]. Indeed, this could be due to the intrinsic nature of pLGG tumors, which in general present lower aggressiveness, slower growth rates, as well as reduced infiltrative capacity. Currently, reliable models for developing new drugs and studying pLGG progression remain limited. Over the past decade, extensive molecular analyses have revealed that pLGGs are primarily driven by upregulation of the RAS/MAPK pathway, highlighting the critical role of frequent activation of both RAS/MAPK and mTOR signaling in their tumorigenesis and progression. Somatic mutations in *BRAF* and germline mutations in *NF1* have been identified as key contributors to tumorigenesis being almost exclusively a single-event driver [12]. Low-grade gliomas frequently exhibit alterations in *BRAF*, a gene encoding a serine/threonine kinase that serves as a downstream regulator of the MAPK pathway. Two common *BRAF* alterations include the oncogenic *BRAF*^*V600E*^ mutation and a fusion between *BRAF* and the large, functionally uncharacterized gene *KIAA1549*. Specifically, *BRAF*^*V600E*^ mutation, *KIAA1549::BRAF* translocations, and *NF1* mutations together account for over 60% of pLGG cases [12]. Interestingly, Anastasaki et al [13]. engineered human induced pluripotent stem cells (hiPSCs) to carry either *NF1* loss or the *KIAA1549::BRAF* fusion and they successively differentiated these hiPSCs into 2D neural stem cells. In the current study, we employed human forebrain organoids to model pLGG, focusing on *BRAF* alterations, and enhanced cellular complexity. We then characterized these models using DNA methylation profiling and single-cell RNA sequencing (scRNA-seq).

## Results

### Generation of human pLGG organoid models

**Fig. 1:**
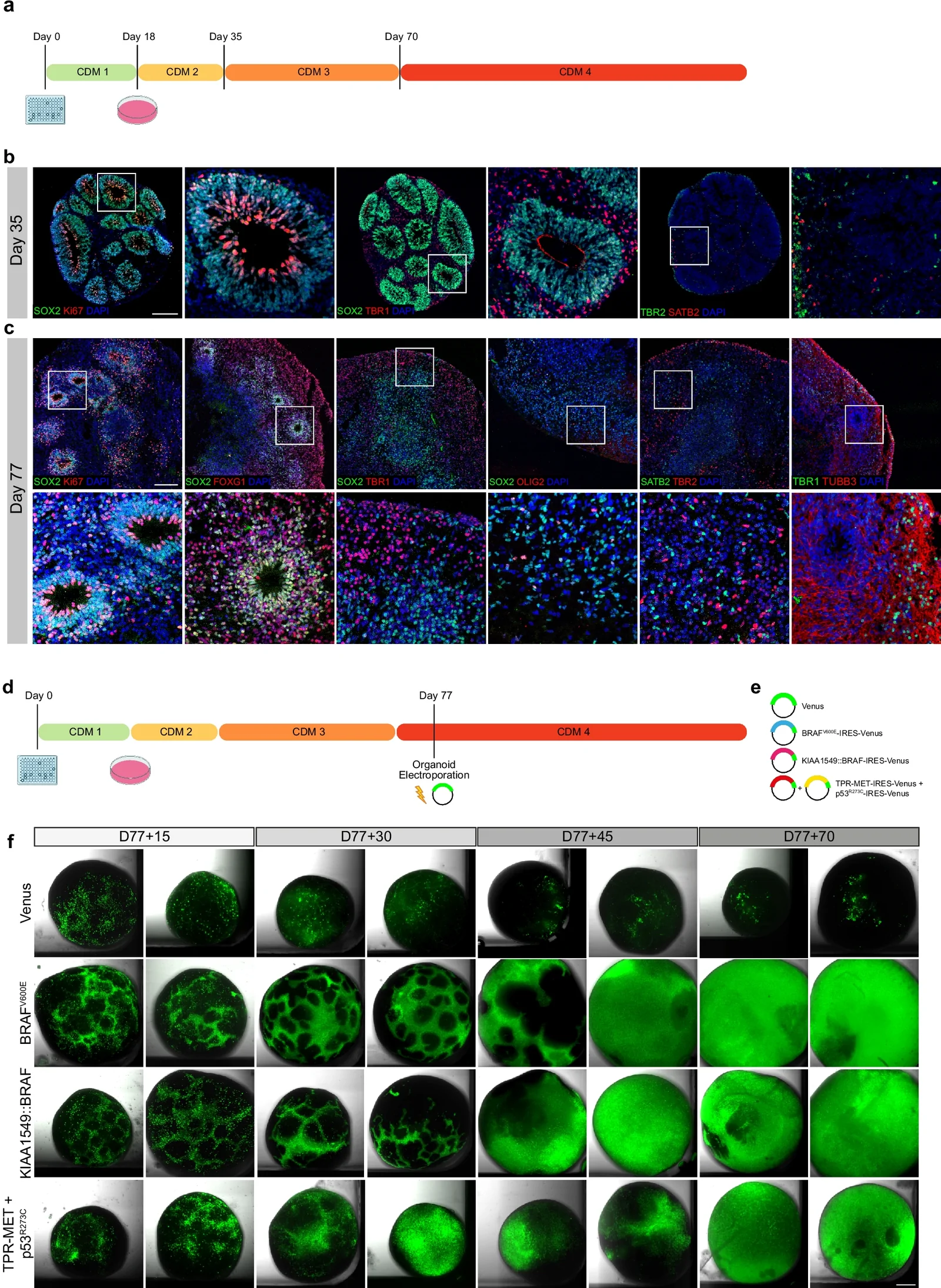
Generation of human pLGG organoid models. **a** Schematic representation of the protocol to generate forebrain organoids (adapted from Velasco et al., 2019); CDM, cortical differentiation medium.** b** Representative images of forebrain organoids at day 35 of differentiation characterized by immunofluorescence for SOX2, Ki67, TBR1, TBR2, SATB2.** c** Representative images of forebrain organoids at day 77 of differentiation characterized by immunofluorescence for SOX2, Ki67, FOXG1, OLIG2, TBR1, TBR2, SATB2, TUBB3.** d**, **e** Schematic representation of the protocol (**d**) and of the genetic combinations (**e**) used for organoids electroporation at day 77 of differentiation.** f** Live imaging of forebrain organoids electroporated at day 77 of differentiation with Venus, *BRAF*^*V600E*^, *KIAA1549::BRAF*, or *TPR-MET* + *p53*^*R273C*^. Organoids were imaged at 15 (D77 + 15), 30 (D77 + 30), 45 (D77 + 45) and 70 (D77 + 70) days post electroporation. Scale bars (**b**, **c**) 200 µm, (**f**) 500 µm

**Fig. 2:**
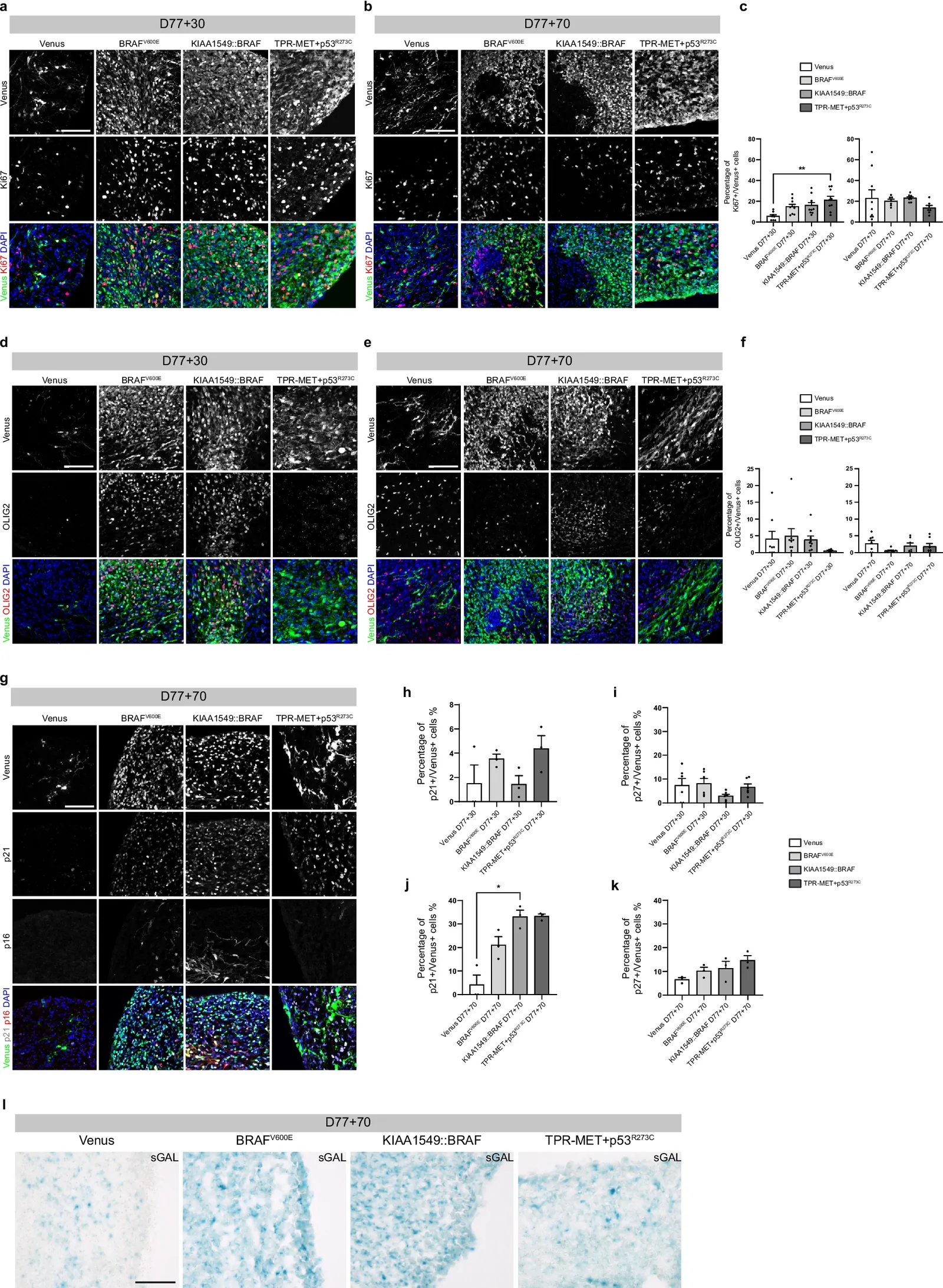
*BRAF*^*V600E*^ and *KIAA1549::BRAF* overexpression induce senescence markers expression. **a**,** b** Confocal images of immunofluorescence of Venus and Ki67 in hiPSC-derived dorsal forebrain organoids electroporated with Venus, *BRAF*^*V600E*^, *KIAA1549::BRAF*, *TPR-MET* + *p53*^*R273C*^ at day 77 + 30 (**a**) and 77 + 70 of differentiation (**b**). **c** Quantifications of Ki67^+^ cells co-expressing Venus in hiPSC-derived dorsal forebrain organoids at day 77 + 30 and at day 77 + 70 of differentiation electroporated with Venus, *BRAF*^*V600E*^, *KIAA1549::BRAF*, *TPR-MET* + *p53*^*R273C*^. **d**, **e** Confocal images of immunofluorescence of Venus and OLIG2 in hiPSC-derived dorsal forebrain organoids electroporated with Venus, *BRAF*^*V600E*^, *KIAA1549::BRAF*, *TPR-MET* + *p53*^*R273C*^ at day 77 + 30 (**d**) and 77 + 70 of differentiation (**e**). **f** Quantifications of OLIG2^+^ cells co-expressing Venus in hiPSC-derived dorsal forebrain organoids at day 77 + 30 and at day 77 + 70 of differentiation electroporated with Venus, *BRAF*^*V600E*^, *KIAA1549::BRAF*, *TPR-MET* + *p53*^*R273C*^. **g** Confocal images of immunofluorescence of Venus, p21, and p16 in hiPSC-derived dorsal forebrain organoids electroporated with Venus, *BRAF*^*V600E*^, *KIAA1549::BRAF*, *TPR-MET* + *p53*^*R273C*^ at day 77 + 70 of differentiation. **h**, **j** Quantifications of p21^+^ cells co-expressing Venus in hiPSC-derived dorsal forebrain organoids at day 77 + 30 (**h**) and 77 + 70 (**j**) of differentiation electroporated with Venus, *BRAF*^*V600E*^, *KIAA1549::BRAF*, *TPR-MET* + *p53*^*R273C*^. **i**, **k** Quantifications of p27^+^ cells co-expressing Venus in hiPSC-derived dorsal forebrain organoids at day 77 + 30 (**i**) and 77 + 70 (**k**) of differentiation electroporated with Venus, *BRAF*^*V600E*^, *KIAA1549::BRAF*, *TPR-MET* + *p53*^*R273C*^. **l** Senescence-associated β-galactosidase (sGAL) staining in hiPSC-derived dorsal forebrain organoids at day 77 + 70 of differentiation electroporated with Venus, *BRAF*^*V600E*^, *KIAA1549::BRAF*, *TPR-MET* + *p53*.^*R273C*^. Scale bars (**a**, **b**, **d**, **e**, **g**, **l**) 100 μm. Data are presented as mean ± S.E.M.; each dot represents a single organoid. For each marker, *n* = 2–5 images were considered. Statistics: Brown-Forsythe and Welch ANOVA tests; *p ≤ 0.05; **p ≤ 0.01; ***p ≤ 0.001

Given that 27% of pLGGs arise in the cerebral hemispheres, we aimed to develop a disease-relevant model. The *BRAF*^*V600E*^ mutation has been detected in 15–20% of pLGG cases [14], predominantly occurring in pleomorphic xanthoastrocytoma, pilocytic astrocytoma, and ganglioglioma subtypes [15]. To recapitulate these conditions, we employed a well-established protocol for generating human forebrain organoids from iPSCs [16] (Fig. 1a). Interestingly, human iPSC-derived forebrain organoids contain human brain progenitor populations that express key stem cells markers at levels comparable to those found in fetal human brain progenitors. These include genes specific for dorsal forebrain progenitors (*FOXG1*, *EMX1* and *PAX6*), intermediate progenitors (*TBR2*), outer radial glia (*HOPX*), and glial progenitors (*GFAP*, *S100b*) [16, 17]. Notably, these neural progenitors can generate neurons expressing markers characteristic of deeper layer cortical neurons, such as *TBR1*, and callosal projected neurons, including *SATB2*. Recently, we have successfully recapitulated forebrain organoid generation to establish an in vitro model of pediatric high-grade glioma [18]. Here, we used the same protocol for forebrain organoids generation and, to mimic pLGG genetic alterations, we used two vectors expressing *BRAF*^*V600E*^ and the *KIAA1549::BRAF* fusion gene in forebrain progenitors (stable expression using the PiggyBac system). Since the pLGG cell of origin has not been clearly identified, we chose two different timepoints (day 35 and day 77 of differentiation) using cortical forebrain progenitors capable of generating distinct cortical neuron subtypes. At day 35 of differentiation, we found mainly SOX2^+^ radial glia, a few TBR2^+^ intermediate progenitors and TBR1^+^/TUBB3^+^ deeper layer neurons (Fig. 1b), while at day 77 we observed an increased number of TBR2^+^ progenitors and more SATB2^+^ neurons (Fig. 1c). Interestingly, we also detected a small population of OLIG2^+^ cells at day 77 (Fig. 1c), suggesting the presence of oligodendrocytes lineage cells within the forebrain organoids, consistent with previous reports [19]. We used two vectors expressing either *BRAF*^*V600E*^ or the translocated gene *KIAA1549::BRAF*. Both plasmids also encoded for the fluorescent protein Venus (IRES Venus, Fig. 1d, e), to track oncogene expressing cells. The vectors have been electroporated in forebrain organoids at day 35 and day 77 of differentiation (Fig. 1d, e and S1a,b). As shown in Fig. 1f and S1c, we observed the presence of Venus^+^ cells 15 days after electroporation. Interestingly, we observed a progressive increase in Venus^+^ cells from 15 to 70 days post-electroporation (D77 + 70) in organoids overexpressing *BRAF*^*V600E*^ or *KIAA1549::BRAF* compared to controls overexpressing only the reporter Venus. At 70 days post-electroporation, the organoids were almost entirely populated by Venus^+^ oncogene-expressing cells. These results were comparable with an organoid model of pediatric high-grade glioma overexpressing TPR-MET and dnTP53 (TP organoids [18]). We then further characterized the organoids by staining for proliferating cells (Ki67^+^), and oligodendrocyte progenitors (OLIG2^+^). As shown in Fig. 2a-c and S2a,c the TP organoids display higher percentage of Ki67^+^ cells compared to control organoids (Venus) at 30 days post electroporation (D77 + 30), whereas *BRAF*^*V600E*^ and *KIAA1549::BRAF*-overexpressing organoids did not show significantly increased number of Ki67^+^ cells. The high percentage of Ki67^+^ cells in the high-grade glioma model reflects the patient's condition. Interestingly, at 70 days post-electroporation, we observed no difference between control organoids and those overexpressing *BRAF*^*V600E*^, *KIAA1549::BRAF* or *TPR-MET* + *dnTP53* (Fig. 2c).

Furthermore, forebrain organoids expressing *BRAF*^*V600E*^ and *KIAA1549::BRAF* displayed presence of OLIG2^+^ cells at levels comparable to those observed in control organoids (Fig. 2d-f and S2b,d), a feature previously reported in pLGG patients [20]. Indeed, astrocytic tumors, including all pilocytic astrocytomas (WHO Grade 1) and diffuse-type astrocytomas (WHO Grades 2–4) showed widespread OLIG2 expression [20]. Interestingly, by examining quiescence markers, we observed that *BRAF*-driven forebrain organoids, expressing *KIAA1549::BRAF*, showed several p21^+^ and p27^+^ cells (Fig. 2g-k). In addition, p16^INK4a^ and sGAL activity, commonly used senescence markers cells, were detected in BRAF organoids while they were almost absent in control organoids (Fig. 2g, l). Collectively, our findings indicate that forebrain organoids with upregulated BRAF signaling display features reminiscent of those observed in pLGG patients.

### *In vivo* grafting of human pLGG organoids mimic patients’ tumors

**Fig. 3:**
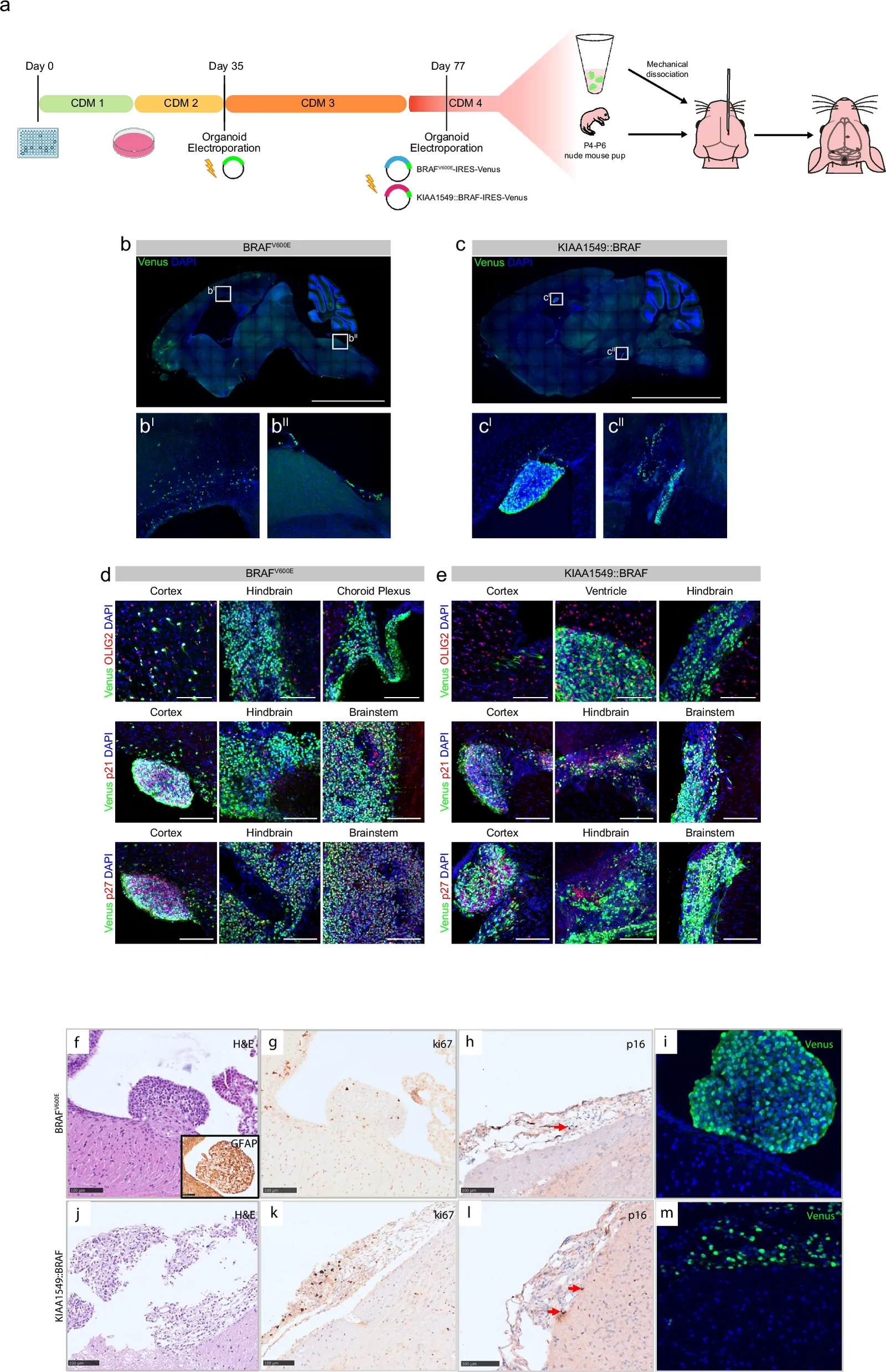
In vivo orthotopic transplantation of human pLGG organoids mimic patient tumors. **a** Schematic representation of forebrain organoids engrafted at day 77 + 80 into the mouse right ventricle at postnatal day 4–6 (P4-P6).** b**, **c** Mosaic image of mouse brain engrafted with forebrain organoids electroporated with *BRAF*^*V600E*^ (**b**) or *KIAA1549::BRAF* (**c**) at day 77 of differentiation. Clusters of Venus^+^ cells are highlighted in the insets (**b’**, **b**’’, **c**’, **c**’’). **d**, **e** Representative images of OLIG2, p21 and p27-expressing cells in brain cryosections of immunodeficient mice engrafted with *BRAF*^*V600E*^ (**d**) or *KIAA1549::BRAF* organoids (**e**). **f** Intraventricular low-grade glioma tumor mass composed of cells with mild nuclear pleomorphism and abundant eosinophilic cytoplasm (H&E—200X). The astrocytic nature of the cells is demonstrated by the GFAP immunostaining (inset – 400X). **g**, **k** IHC of Ki67 on mouse brain FFPE sections derived from engraftment of *BRAF*^*V600E*^ (**g**) or *KIAA1549::BRAF* organoids (**k**), showing low proliferation index (200X). **h**, **l** IHC of p16 on mouse brain FFPE sections derived from engraftment of *BRAF*^*V600E*^ (**h**) or *KIAA1549::BRAF* organoids (**l**). **i**, **m** Representative immunofluorescence images of mouse brain FFPE sections derived from engraftment of *BRAF*^*V600E*^ (**i**) or *KIAA1549::BRAF* organoids (**m**) (100X). Neoplastic Venus^+^ cells are observed forming small clusters adhering to healthy mouse brain tissue, with no evidence of infiltration, vascular invasion, or involvement of cerebellar spaces. **j** Subpial low grade glioma (H&E – 200X). Scale bars (**b**, **c**) 5 mm, (**d**-**m**) 100 µm

To further characterize our pLGG models at day 77 + 70, we tested their capacity in generating tumors after ventricle/cortex transplantation in immunodeficient mice (Fig. 3a). We injected two *BRAF*^*V600E*^-electroporated organoids into 7 Foxn1^nu/nu^ mice and two *KIAA1549::BRAF*-electroporated organoids into 8 Foxn1^nu/nu^ mice. Mice brains were analyzed three months after grafting, as no signs of morbidity or suffering were detected before experimental endpoint. Interestingly, we observed the formation of small clusters of Venus^+^ electroporated cells (Fig. 3b, c) with both *BRAF*^*V600*E^ and *KIAA1549::BRAF* modified organoids. The engrafted cells express *BRAF*^*V600*E^ (Figure S3a,b), OLIG2, p21, p27 (Fig. 3d, e), Ki67 and p16 (Figure S3c-e) markers. The histological examination revealed neoplastic lesions in all cases analyzed, except one (Figure S3f, mouse 3c). Neoplastic cells exhibited either subdural or intraventricular growth patterns (Fig. 3f, j). All cases were positive for GFAP, indicating astrocytic differentiation (Fig. 3f and Figure S3f). Two cases showed rare synaptophysin positive cells (Figure S3f). The Ki67 values corresponded to the high- (> 10%) or low-grade features of the tumors (Fig. 3g, k and Figure S3f). Among the engraftments with organoids carrying the *BRAF*^*V600E*^ mutation, all cases showed positivity for both immunohistochemical and immunofluorescence staining for Ki67. Six tumors showed low-grade features, while one exhibited slightly increased cellularity associated with an infiltrative pattern of the neoplasm involving brain parenchyma and vasculature, as well as a higher Ki67 index compared to other cases. Among the engraftments with organoids harboring the *KIAA1549::BRAF* fusion, four exhibited a low-grade glioma morphology, two exhibited high-grade characteristics, and one displayed intermediate-grade traits (Figure S3f). p16^+^ cells were detected in 10 tumors (Fig. 3h, i, l, m and Figure S3e), confirming the presence of senescent cancer cells also in vivo. As control, we also grafted wildtype forebrain organoids in nude mice, and in this case we observed human cells in proliferation (Figure S3g,h), with the organoid’s mass not infiltrating the brain parenchyma. Overall, our data suggest that the *BRAF* organoid models, when grafted in vivo, recapitulate several key features of pLGG. This stands in contrast with our previous pediatric high-grade glioma (HGG) model (TP organoids), in which hiPSC-derived tumors exhibited rapid growth, infiltrative behavior *in vivo* [18] and p21^+^ and p16^+^ cells (Figure S3i). On the contrary, our *BRAF* organoid models appear to generate smaller and less aggressive tumors.

### Molecular classification of human pLGG organoid models

**Fig. 4:**
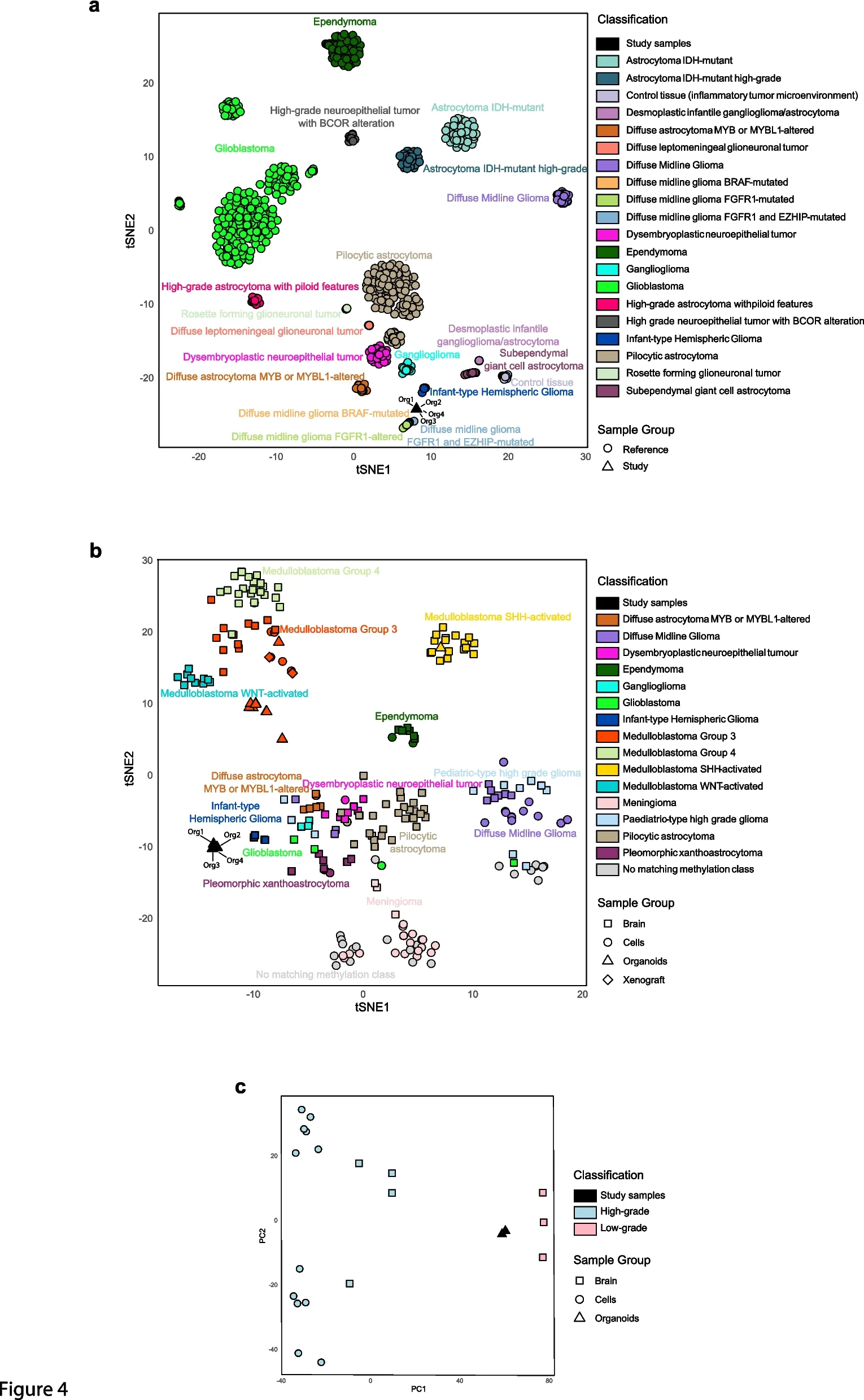
Molecular Classification of human pLGG organoid models. **a** t-SNE analysis of DNA methylation data comparing four *BRAF*^*V600E*^ and *KIAA1549::BRAF*-expressing organoids (black) to a broad brain tumor dataset (from Auffret et al.) consisting of 997 brain tumor samples (colored by methylation class). Sample cohort is indicated by point shape: triangles represent study samples, while circles represent reference samples. **b** t-SNE analysis of DNA methylation data comparing four *BRAF*^*V600E*^ and *KIAA1549::BRAF*-expressing organoids (black) to a pediatric brain tumor and cellular model dataset of 232 reference samples (226 described by Pedace et al.), comprising 149 brain tumors and 76 cellular models. Six additional cellular models and one brain tumor tissue were included. Points are colored by methylation class, with shape indicating sample type: squares for brain tumor tissues, circles for cell lines, triangles for organoids, and diamonds for xenografts. **c** PCA analysis of transcriptomic data comparing *BRAF*^*V600E*^ and *KIAA1549::BRAF*-expressing organoids (black) to pediatric low-grade and high-grade gliomas and cellular models**.** The analysis includes two study samples (black) and 19 reference samples. The reference group consists of 3 unpublished low-grade glioma samples, 4 high-grade tumor tissues, and 12 high-grade cell models previously described [21]. Points are colored by histological classification (light pink for low-grade gliomas, light blue for high-grade gliomas), and shapes indicate sample type: squares for brain tumor tissues, circles for cell lines, and triangles for organoids

To further validate our pLGG models, we analyzed the DNA methylation profiles of the organoids grafted in immunodeficient mice. Using the Heidelberg Brain Tumor Classifier, all study samples were assigned to the DNA methylation class *Infant-type Hemispheric Glioma* (IHG), with high calibration scores (> 0.8), indicating a robust and reproducible epigenetic classification. To further contextualize these findings and to address complementary biological questions, we performed t-SNE analyses using two independent reference datasets. The first dataset consisted exclusively of pediatric brain tumor tissue samples [21] and was designed to position the organoid models within the landscape of established tumor entities. In this analysis, the study samples formed a compact and distinct cluster that did not fully overlap with any single reference class (Fig. 4a, triangles), indicating that the models capture a coherent tumor-related epigenetic identity while remaining distinct from canonical tumor tissues. Importantly, the organoids localized preferentially within the spectrum of low-grade glioma entities rather than high-grade tumors, consistent with the biological focus of this study on pediatric low-grade gliomas. Within this context, the closest epigenetic proximity was observed to Infant-type Hemispheric Gliomas, in agreement with the Heidelberg classifier results. Although IHGs are classified as high-grade tumors, they are known to exhibit marked biological heterogeneity and may present with low-grade morphology in a subset of cases. Moreover, even in histologically high-grade presentations, IHGs generally show a more favorable clinical outcome compared with other pediatric high-grade gliomas. These features place IHGs at the interface between low- and high-grade pediatric gliomas, providing a biologically plausible explanation for their proximity to pLGG-derived models. Notably, the organoid samples also localized near BRAF-mutant diffuse midline gliomas in the t-SNE space (Fig. 4a). While these entities do not form a shared cluster, their spatial proximity reflects partial overlap in epigenetic features rather than direct classification equivalence. This observation is consistent with the presence of engineered MAPK pathway alterations in the organoid models and suggests that oncogenic BRAF signaling can induce convergent epigenetic programs across distinct pediatric glioma subtypes. The second reference dataset [22] included pediatric brain tumor tissues together with previously published cellular models and was specifically used to assess model fidelity. In this analysis, the organoid models again formed a discrete cluster and, importantly, did not group with cellular models previously reported to be epigenetically unfaithful (Fig. 4b, circles). This finding supports the robustness of the organoid system in preserving tumor-relevant DNA methylation patterns. Whole-transcriptome gene expression profiling revealed that the organoid samples closely resembled pediatric low-grade glioma tissues, distinctly separating them from pediatric high-grade gliomas [23] (Fig. 4c). DNA methylation-based copy number variation analysis of each sample (Figure S4a-d) revealed that each of the characterized organoid had a flat profile, with no indication of chromosomal changes, which is consistent with a low-grade histology. Analysis of the differentially expressed genes (DEGs) yielded 8200 DEGs between high- and low-grade tumor tissues and 10,193 between high- and low- grade cell models. 5949 genes were shared between these two comparisons, suggesting that a common deregulation pattern differentiated the low-grade tissue and cell models from their high-grade counterparts (Figure S4e). From pathway enrichment analysis, several pathways related to cell senescence and the MAPK gene program emerged (Figure S4f).

### pLGG organoid cells align with OC-like, AC-like and MAPK signature clusters

**Fig. 5:**
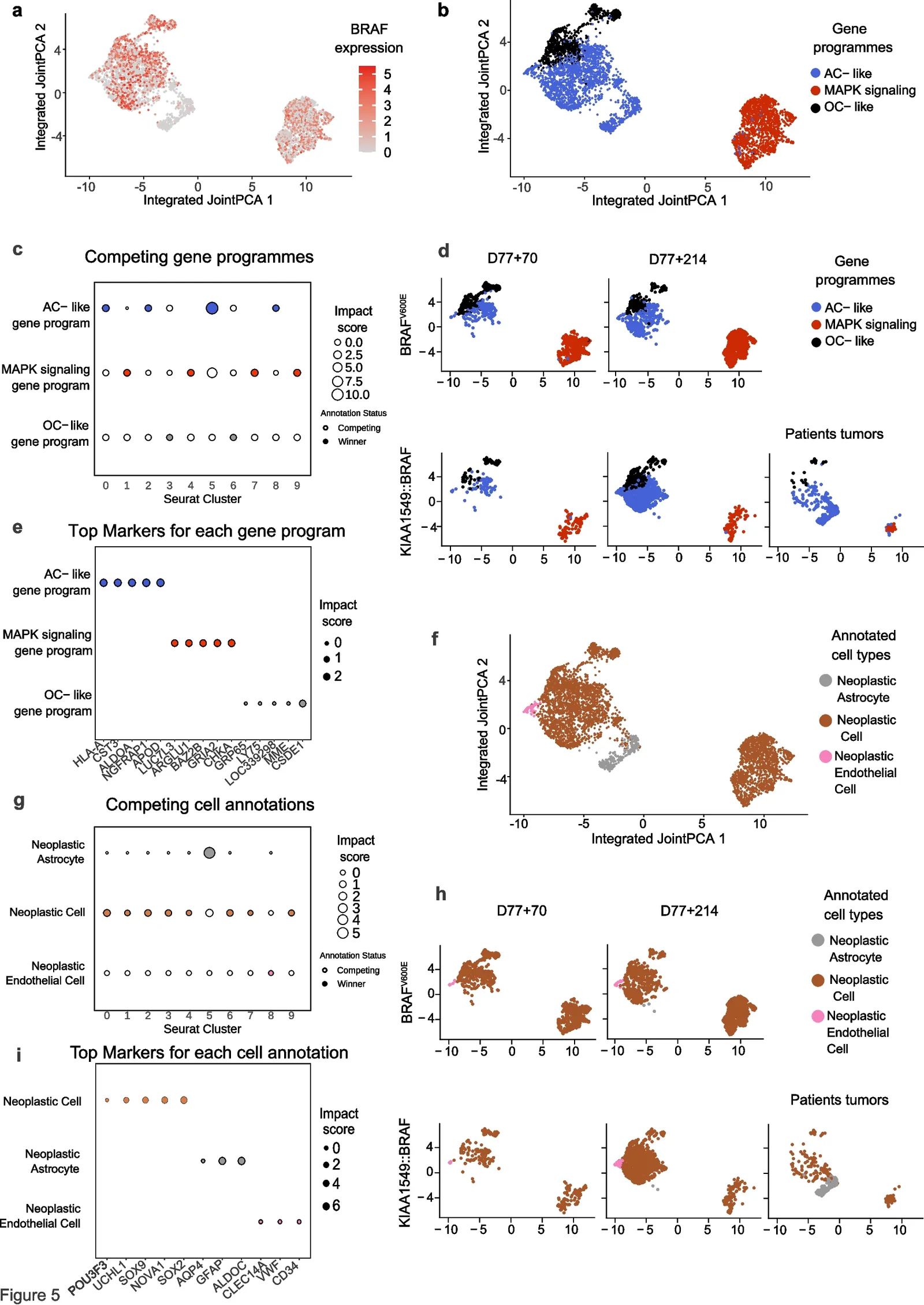
pLGG organoid cells align with OC-like, AC-like and MAPK signature clusters. **a** UMAP visualization of scRNA-seq data where each point represents a single cell. Cells are colored according to the log-normalized expression level of the *BRAF* gene, with red indicating high expression and grey indicating low or no expression. **b** UMAP visualization of scRNA-seq data where each point represents a single cell. Cells are colored by gene programs, using markers from Supplementary Data 6 from Reitman et al., 2019. **c** Dot plot showing the competing gene programmes for each Seurat cluster. Dot size is proportional to the impact score. For each cluster, the gene program that was ultimately assigned as the characteristic signature is indicated by a filled dot (winner annotation), white otherwise. The colors indicate the gene program as in the UMAP visualizations. **d** UMAP visualizations of scRNA-seq data where each point represents a single cell divided by sample type and timepoint of pLGG organoids (*BRAF*^*V600E*^ and *KIAA1549::BRAF*-expressing organoids at D77 + 70, *n* = 1 organoid per sample type, and at D77 + 214, *n* = 3 organoids per sample type) and samples of patient-derived tumors. Colors indicate gene programs. **e** Dot plot identifying the top marker genes that define each gene program. Dot size is proportional to the impact score. The specific marker genes are listed along the x-axis. **f** UMAP visualization of scRNA-seq data where each point represents a single cell. Cells are colored by cell annotation, using markers from cellMarkerAccordion. **g** Dot plot showing the competing cell annotation for each Seurat cluster. Dot size is proportional to the impact score. For each cluster, the cell type that was ultimately assigned as the characteristic signature is indicated by a filled dot (winner annotation), white otherwise. The colors indicate the cell annotation as in the UMAP visualizations. **h** UMAP visualizations of scRNA-seq data where each point represents a single cell divided by sample type and timepoint of pLGG organoids (*BRAF*^*V600E*^ and *KIAA1549::BRAF*-expressing organoids at D77 + 70, *n* = 1 organoid per sample type, and at D77 + 214, *n* = 3 organoids per sample type) and samples of patient-derived tumors. Colors indicate cell annotation. **i** Dot plot identifying the top marker genes that define each cell annotation. Dot size is proportional to the impact score. The specific marker genes are listed along the x-axis. Data from all samples were computationally integrated using the JointPCA method to correct for technical batch effects

To further analyze our organoids we performed single-cell RNA sequencing (scRNA-seq) at an early timepoint (D77 + 70) and a late one (D77 + 214) to detect changes in the organoids’ cell populations after a long time in culture. Interestingly, organoids at later timepoints are almost completely composed of Venus^+^ cells, (Fig. 1f and Figure S5a-d) and express comparable levels of Ki67, p27 and OLIG2 to those observed in earlier organoids (Figure S5e-h). We compared our models’ transcriptional profiles with those of six pilocytic astrocytomas (PA) harboring the *KIAA1549::BRAF* translocation [24]. Interestingly, our pLGG organoid-derived cells clustered closely with patient-derived PA cells, independently of the time point or the expression of *KIAA1549::BRAF* or *BRAF*^*V600E*^, suggesting that both organoid models give rise to comparable cellular populations (Fig. 5a, b, Figure S6a-j). To further characterize the cell populations, we performed functional gene enrichment analysis on cluster-specific positive markers. This reveals distinct cellular programs, characterizing Cluster 2 by oxidative phosphorylation and ATP synthesis and Cluster 6 by cell division (Table S1-3, Figure S7a,b). PA cancer cells recapitulated a developmental differentiation hierarchy, progressing from oligodendrocyte-like (OC-like) states to mature astrocyte-like (AC-like) phenotypes, and exhibited heterogeneous expression of the MAPK signaling gene program (Fig. 5c, d) [24]. Notably, our pLGG organoid cells aligned with OC-like, AC-like and MAPK signature clusters, indicating that the organoids contain a mixed population of cancer cells (Fig. 5c-e). Interestingly, these transcriptional programs segregate into two distinct macro-compartments within the UMAP space (one defined by AC-like/OC-like signature and the other by MAPK signature), hence further investigation using gene enrichment analyses showed differences in cellular and aerobic respiration (Figure S7c,d, Table S4-6). This represents a significant advancement, as hiPSC-derived cancer organoids are typically thought to produce relatively homogeneous cell populations. In contrast, our model captures three distinct cancer subpopulations, closely resembling the cellular heterogeneity observed in patients (Fig. 5c, d).

We then performed cell type annotation using high- and low-grade glioma markers from the Cell Marker Accordion package [25]. Three populations were identified: neoplastic astrocyte, neoplastic cells and neoplastic endothelial cells. Interestingly, the neoplastic astrocyte population corresponds to cluster 5 (Fig. 5f-h, S6b). Moreover, cellular heterogeneity is preserved in the organoids cells, since organoids cells are classified both as Neoplastic Cell and Neoplastic Endothelial cells (Fig. 5h, i, S6i). Finally, an analysis of cell distribution across the different timepoints and different samples demonstrated that cells from each individual organoid sample, regardless of mutation or culture duration, contributed to all identified gene programs and annotated cell types (Figure S6f-j).

### Tovorafenib in vitro testing

To evaluate whether our new hiPSC-derived pLGG models are suitable for drug testing, we employed tovorafenib, a drug previously tested in a clinical trial involving patients with *KIAA1549::BRAF* translocations (NCT04775485). This trial demonstrated that tovorafenib induces clinically meaningful tumor responses in children and young adults with *BRAF*-altered relapsed/refractory pLGG [6].

**Fig. 6:**
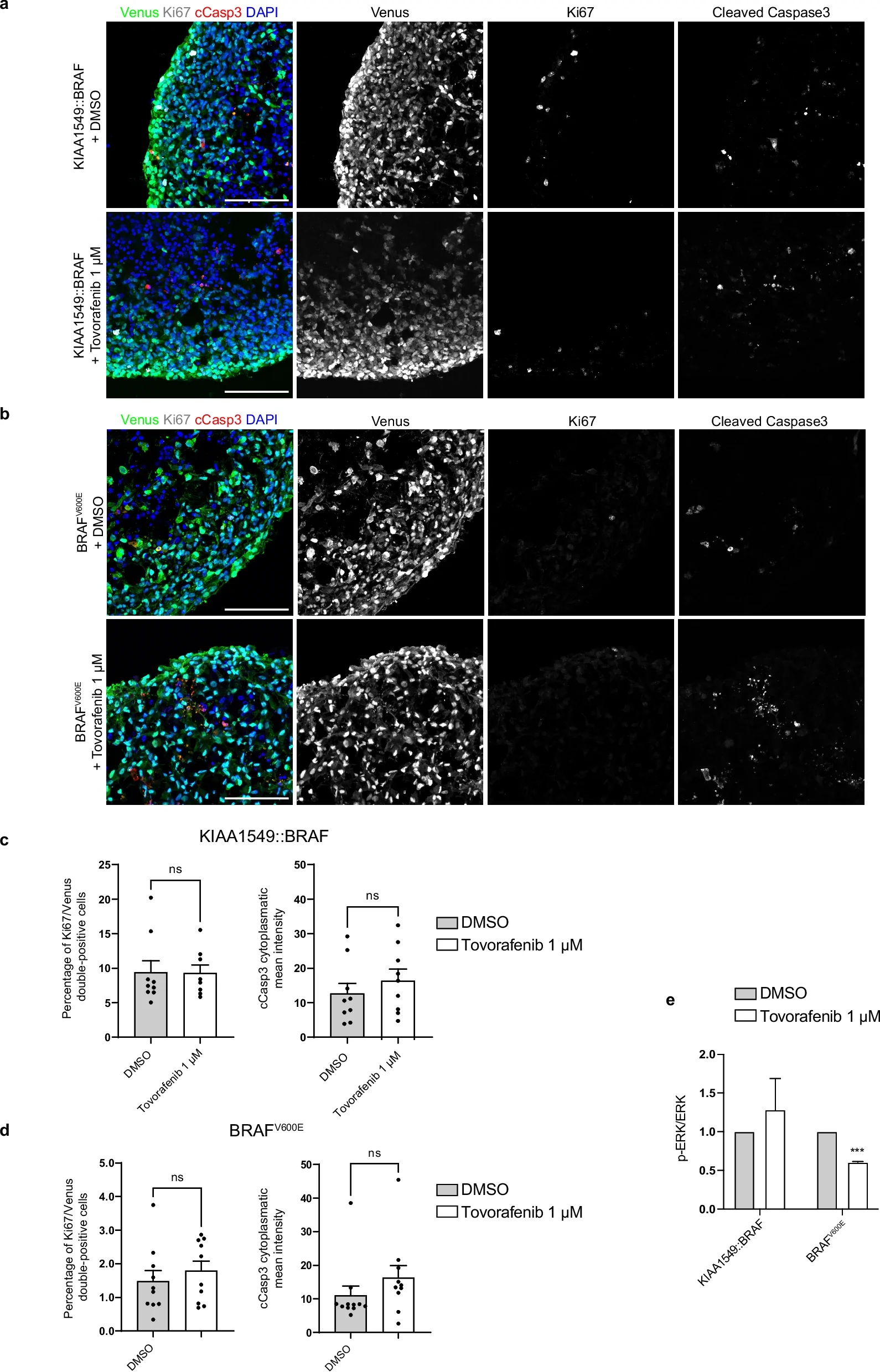
Tovorafenib does modulate cell proliferation or apoptosis in *BRAF*^*V600E*^ and *KIAA1549::BRAF* organoids. **a**, **b** Confocal images of immunofluorescence of Venus, Ki67, Cleaved Caspase 3 in hiPSC-derived dorsal forebrain organoids electroporated with *KIAA1549::BRAF* (**a**) and *BRAF*^*V600E*^ (**b**), treated with tovorafenib 1 µM or DMSO (vehicle). **c**, **d** Quantifications of Ki67^+^ cells co-expressing Venus, and quantification of Cleaved Caspase 3 cytoplasmic signal in hiPSC-derived dorsal forebrain organoids at day 77 + 300 or 77 + 320 of differentiation electroporated with *KIAA1549::BRAF* (**c**) or *BRAF*^*V600E*^ (**d**), treated with tovorafenib 1 µM or DMSO (vehicle). Data are presented as mean ± S.E.M.; each dot represents a single organoid. For each marker, *n* = 3–4 images of electroporated organoids were considered. Statistics: Mann–Whitney test for data with non‐normal distribution or parametric two-tailed T-test with Welch’s correction for data with normal distribution; *p ≤ 0.05; **p ≤ 0.01; ***p ≤ 0.001. Scale bar (**a**, **b**) 100 μm. **e** Western blot analysis showing the normalized expression of phosphorylated ERK (p-ERK) on total ERK in hiPSC-derived dorsal forebrain organoids at day 77 + 300 or 77 + 320 of differentiation, electroporated either with *KIAA1549::BRAF* or *BRAF*.^*V600E*^ and treated with tovorafenib 1 µM or DMSO (vehicle). Densitometric values of pERK were normalized to total ERK, and the resulting ratios were normalized to control. Ponceau staining was used as loading control. Statistics: two-tailed Student’s t-test between tovorafenib treatment and control (vehicle); *p ≤ 0.05; **p ≤ 0.01; ***p ≤ 0.001

We tested tovorafenib on 77 + 300-day-old pLGG organoids electroporated at day 77 with either *KIAA1549::BRAF* or *BRAF*^*V600E*^, as described previously. Notably, in the absence of treatment, these late-stage organoids exhibited lower levels of Ki67^+^ cells (Fig. 6a-d) compared to earlier timepoints and HGG organoids (TP organoids) (Fig. 2c). Thus, these mature organoids may provide a more physiologically relevant model for assessing tovorafenib’s effects on cell survival and proliferation, better recapitulating the clinical setting.

Interestingly, following tovorafenib treatment no significant changes in either cell death or proliferation were observed (Fig. 6a-d). A modest decrease in ERK phosphorylation (p-ERK) was detected in *BRAF*^*V600E*^-electroporated organoids, whereas no change was detected in those electroporated with *KIAA1549::BRAF* (Fig. 6e, S8a). As control for the tovorafenib treatment, we used a melanoma cell line that responds to the tovorafenib treatment (Figure S8b-d).

These findings indicate that our pLGG model did not respond to tovorafenib under the tested conditions, potentially due to limited sensitivity, insufficient drug penetration, or compound instability in the culture medium. Therefore, this pLGG model may serve as a valuable platform for evaluating alternative or next-generation therapeutic strategies.

## Discussion

Our study provides new insights into the molecular and cellular features of BRAF-driven pediatric low-grade glioma (pLGG) models generated from human forebrain organoids. The high proportion of Ki67⁺ cells observed in the high-grade glioma (HGG) organoid model mirrors the proliferative index of patient-derived tumors, underscoring the validity of this platform to recapitulate clinically relevant tumor behavior [18]. In contrast, BRAF-driven forebrain organoids, harboring either *BRAF*^*V600E*^ or *KIAA1549::BRAF* overexpression, exhibited distinct features that align with hallmarks of pLGG. In particular, the presence of OLIG2^+^ cells is consistent with previous reports describing widespread OLIG2 expression in pilocytic astrocytomas (WHO grade 1) and diffuse astrocytomas (WHO grades 2–4) [20]. This reinforces the notion that our organoid-based models capture lineage-specific features of pLGG biology.

In addition to OLIG2 expression, BRAF-driven organoids showed increased numbers of cells positive for quiescence and senescence markers, including, p27, p16 and sGAL activity. This finding is of particular interest, as cellular senescence has been proposed as a tumor-suppressive mechanism that counterbalances oncogene-induced proliferation in pLGG [7, 8]. The detection of p16⁺ cells and sGAL activity in BRAF-altered organoids, but not in controls organoids, suggests that activation of the BRAF pathway induces a growth-arrest program, in line with models of oncogene-induced senescence (OIS) [7, 8]. Furthermore, pathway enrichment analysis revealed that low-grade organoids are characterized by the activation of gene programs associated with cell senescence and MAPK signaling. We therefore propose that our system may be particularly informative for studying early tumorigenic events, a stage that remains poorly understood in both low- and high-grade gliomas. At this early phase, markers such as p21, Ki67, and OLIG2 appear to be expressed at comparable levels, suggesting that the two tumor types may initially share common biological features before diverging as high-grade gliomas acquire additional oncogenic alterations that drive malignant progression. Consistent with this notion, pLGGs typically exhibit few genetic alterations and generally lack mutations in canonical tumor suppressor genes, potentially explaining their relatively low proliferation rates and modulated mitotic activity. However, *BRAF*^*V600E*^ mutations can be associated with additional genomic lesions, such as deletion of the tumor suppressor gene *CDKN2A* (Cyclin dependent kinase inhibitor 2 A), a combination that is thought to contribute to tumor progression and increased malignancy [24].

Another significant finding would be the identification of the pLGG cell of origin. Human iPSC-derived forebrain organoids contain multiple neural populations, including dorsal forebrain progenitors (expressing *FOXG1*, *EMX1*, and *PAX6*), intermediate progenitors (*TBR2*), outer radial glia (*HOPX*), and glial progenitors (*GFAP* and *S100b*), together with a small subset of OLIG2^+^ cells [16, 17]. Future studies should exploit cell type–specific promoters and temporally controlled genetic manipulation to identify which of these populations are competent to initiate pLGG formation, using strategies similar to those previously developed for medulloblastoma modeling [26–28]. Recently, reported 2D systems based on engineered iPSCs modeling *NF1* loss or *KIAA1549::BRAF* fusion provide additional support for the feasibility of this approach, although they lack the regional and cytoarchitectural specificity offered by brain organoids [13]. Extending our strategy to midbrain and hindbrain organoids will be essential to explore the tumorigenic potential of other progenitor populations and to better reflect the anatomical diversity of pLGGs.

At the single-cell level, our pLGG organoid-derived cells align with the OC-like, AC-like and MAPK signature clusters previously identified in patient tumors, indicating that these organoids give rise to a heterogeneous population of cancer cells. This observation is particularly relevant, as intratumoral heterogeneity is a defining feature of pLGGs and is thought to underlie differences in tumor behavior, therapeutic response, and resistance mechanisms. While hiPSC-derived cancer organoids have often been considered relatively homogeneous, our model captures at least three transcriptionally distinct cancer cell states that mirror those found in patients, including populations with OC-like and AC-like features and those characterized by strong MAPK pathway activation. This degree of cellular diversity underscores the value of the model for investigating pLGG pathogenesis, although it should currently be regarded as an exploratory and hypothesis-generating platform rather than a fully validated system for predictive drug screening.

When assessing the translational potential of our models, we evaluated the response to tovorafenib, a type II pan-RAF inhibitor recently tested in patients with BRAF-altered pLGG. In the phase II trial [6], the overall response rate (ORR), including minimal responses according to RAPNO and RANO-LGG criteria, was approximately 51–55%. Comparable response rates have also been reported in optic pathway gliomas [29] and in small cohorts of adult HGG and LGG patients [30]. These data indicate that nearly half of patients do not achieve an objective radiological response, highlighting that intrinsic or early resistance to tovorafenib is a clinically relevant phenomenon.

Our organoid models did not exhibit sensitivity to tovorafenib, showing no significant effects on proliferation or apoptosis. Only a modest reduction in p-ERK levels was detected in *BRAF*^*V600*E^-expressing organoids, with no measurable impact in those harboring *KIAA1549::BRAF*. Importantly, clinical responses to tovorafenib are typically delayed: in the phase II study, the median time to initial response (minimal or partial response) was 5.5 months overall, reaching up to 11.3 months in some patients, while the median duration of response was 14.4 months and the median progression-free survival was 13.9 months [6]. Such prolonged kinetics are inherently difficult to recapitulate in short-term in vitro assays, which usually assess drug effects over days to a few weeks. Therefore, the absence of an early measurable response in our organoid system does not necessarily contradict clinical efficacy but rather could reflect fundamental differences between acute in vitro drug exposure and long-term in vivo tumor dynamics.

Moreover, given that only about half of patients respond clinically, it is plausible that our hiPSC-derived and engineered organoid model mirrors the non-responder patient fraction. In this context, the lack of sensitivity observed in our model may represent a biologically meaningful feature rather than a technical failure, potentially providing a valuable platform to investigate mechanisms of intrinsic resistance, identify biomarkers predictive of response, and explore rational combination therapies aimed at overcoming therapeutic refractoriness.

At present, our data indicate that the pLGG organoid model should not be considered a standalone predictive platform for drug screening. Instead, it represents an important first step toward the development of more comprehensive functional models capable of capturing the heterogeneity of patient responses. Future work should aim to expand beyond engineered hiPSCs models by establishing a panel of pLGG organoids derived directly from multiple patients’ biopsies, ultimately recapitulating the proportions of responders and non-responders tumors observed clinically. This would allow stratification of drug sensitivity patterns and enable a more accurate assessment of translational relevance.

In addition, alternative experimental strategies may further improve the modeling of therapeutic responses. These include modifying treatment schedules to better mimic clinical regimens, extending drug exposure times, and implementing longitudinal assays to capture delayed or adaptive responses. The incorporation of pharmacodynamic readouts, pathway inhibition markers, and cell-state transitions may also reveal subtler biological effects of targeted therapies that are not captured by short-term viability assays.

However, their apparent lack of sensitivity to tovorafenib highlights important limitations and emphasizes that this model currently represents an exploratory and hypothesis-generating platform rather than a fully validated system for predictive drug testing. By explicitly acknowledging these constraints, our work establishes a foundation for future refinements aimed at modeling patient heterogeneity, treatment timing, and long-term drug responses, thereby moving toward more physiologically relevant and clinically informative platforms for pediatric glioma research.

### Limitations of the study

Our pLGG organoid model, generated from human induced pluripotent stem cells, successfully reproduces key features of the three-dimensional organization of the developing human brain. These organoids are capable of recapitulating several salient stages of neurodevelopment and pLGG generation. As a result, they represent a promising platform for studying pediatric low-grade glioma (pLGG) biology in a controlled, human-relevant context. However, despite these advantages, the use of brain organoids to investigate the onset, progression, and underlying mechanisms of pLGG development still faces substantial challenges.

One major constraint is the absence of an immune cell compartment. Microglia and peripheral immune cells play critical roles in shaping the tumor microenvironment, influencing tumor initiation, growth, and response to external stimuli. Without these components, organoids provide only a partial representation of pLGGs biology and may overlook important immune-mediated mechanisms.

A second key limitation is the lack of vascularization. Blood vessels are essential not only for nutrient and oxygen delivery but also for establishing metabolic gradients and cellular interactions that are central to tumor development. The avascular nature of current brain organoids restricts long-term growth, limits maturation, and fails to capture the vascular contributions to pLGGs pathology, including blood–brain barrier dynamics and vessel-tumor interactions.

Together, these shortcomings underscore the need for next-generation organoid platforms that incorporate additional cellular and structural complexity. Future efforts should focus on integrating immune cells and establishing functional vascular networks, potentially through bioengineering, co-culture systems, or transplantation approaches. Enhancing organoid fidelity in this way will be crucial for developing more physiologically relevant models that accurately reflect the multifaceted environment in which pLGGs arise and evolve.

## Material and methods

### Plasmids generation

The hyperactive form of piggyBac transposase (pCMV-Hahy-pBase, pPBase) was donated by the Wellcome Sanger Institute. pPB-CAG-Venus was generated previously [28]. The coding sequence of the oncogenes used in this study was amplified by PCR or digested using restriction enzymes and subsequently cloned into the MCS of pPB-CAG-MCS-ires-Venus. TPR-MET was amplified by PCR from pBABE-puro-TPR-MET (Addgene, 10902), p53 (R273C) was cloned from pCMV-Neo-Bam-p53R273C (a kind gift from Alberto Inga), while BRAF(V600E)-ires-Venus was cloned starting from pBabe-B-Raf(V600E) (Addgene, 17544). To obtain pPB-CAG-KIAA1549::BRAF-ires-Venus, the sequence of *KIAA1549::BRAF* was divided in two inserts through enzymatic digestion with NheI, MluI and SpeI, starting from pLVX-Puro-KIAA1549::BRAF16_9, and subcloned into pPB-CAG-PDGFRα-ires-Venus.

### Mice husbandry

Mice were housed in a certified specific Pathogen Free (SPF) animal facility in accordance with European Guidelines. Mice were housed at 12 h/12 h Dark/Light cycle, 22 °C temperature. The study was carried out in accordance with the recommendations of the European Guidelines. Foxn1^nu/nu^ (086NU/NUCD1, Charles Rivers Laboratories) immunodeficient mice were sacrificed at experimental endpoint or at human endpoint, as they displayed signs of morbidity (loss of weight, loss of coordination, hunched posture).

### Generation and electroporation of human forebrain organoids

Human induced pluripotent stem cells (hiPSCs, a gift from Domenico Delia, IFOM, Milan) were cultured on Geltrex coated plates (Gibco, A1413301) in Essential 8 medium (Gibco, A1517101) supplemented with Penicillin–Streptomycin (P/S; Penicillin 100 Units/ml, Streptomycin 100 μg/ml, Gibco, 15140122). All cells were mycoplasma free. Dorsal forebrain organoids were generated from hiPSCs as previously described [18, 28]. In particular, hiPSCs were dissociated in clumps and seeded at the density of 9,000 cells per well in low-attachment V-bottom 96-well plates for facilitating their aggregation. After 18 days, small organoids were transferred to 100 mm dishes and cultured in agitation until day 35 when electroporation is performed. The addition of TGFβ inhibitor and Wnt inhibitor for the first 18 days promotes the neuronal differentiation toward a telencephalic identity of the forebrain [18, 28]. On day 35 and day 77, organoids were electroporated as previously reported [18, 28]. In particular, 10–15 organoids per condition were electroporated using Gene Pulser Xcell Eukaryotic System (Biorad), following the indications reported in Table 1. The DNA mix contained the piggyBac transposase and the donor plasmids mixed at 1:4 ratio (more details regarding the plasmids used in each oncogenic combination are listed in Table 2), and was diluted in the electroporation buffer [28].

**Table 1:** Information for electroporation setup of forebrain organoids electroporated at day 35 or day 77

	Day 35	Day 77
Voltage (V)	100	200
Pulse length (ms)	10	10
Number of pulses	5	5
Pulse interval (s)	0.1	0.1
Cuvette (mm)	2	4
DNA mix (µg)	100	200
Electroporation buffer (µl)	100	200

**Table 2:** Plasmid composition for the preparation of the electroporation mix

Combination	Plasmid	Ratio
Venus	pCMV-Hahy-pBase	1
	pPB-CAG-Venus	2
	pPB-CAG-LSL-MCS	2
BRAF^V600E^	pCMV-Hahy-pBase	1
	pPB-CAG-BRAF(V600E)-ires-Venus	2
	pPB-CAG-LSL-MCS	2
*KIAA1549::BRAF*	pCMV-Hahy-pBase	1
	pPB-CAG-KIAA1549::BRAF-ires-Venus	4
*TPR-MET* + *p53*^*R273C*^	pCMV-Hahy-pBase	1
	pPB-CAG-TPR-MET-ires-Venus	1,5
	pPB-CAG-p53(R273C)-ires-Venus	1,5
	pPB-CAG-Venus	1

### Orthotopic transplantation of human forebrain organoids in nude mice

Forebrain organoids at 70 days post electroporation (electroporated at day 77, D77 + 70) were transplanted in the right ventricle of Fox1^nu/nu^ immunodeficient mice at postnatal day 5 (P5) as previously described [28] (Charles Rivers Laboratories)**.** Briefly, 2–3 forebrain organoids were dissociated and injected with a 26 s-gauge Hamilton Syringe (80300/00) using a stereotactic apparatus at the following coordinates (from lambda): − 1.5 D/V, + 1.2 M/L, + 1.5 A/P.

### Genomic DNA (gDNA) extraction

Mouse brains engrafted with *BRAF*^*V600E*^*-* or *KIAA1549::BRAF*-overexpressing organoids were freshly dissected and cut in half following the sagittal plane, to allow RNA (see “RNA extraction and RNA sequencing” section) and genomic DNA extraction from the same sample. For gDNA extraction, Venus^+^ tissue was isolated through observation at a fluorescent binocular microscope, snap frozen in liquid nitrogen and stored at − 80 °C. Tumor samples were lysed in lysis buffer (20 mM EDTA, 10 mM Tris, 200 mM NaCl, 0.2% Triton X-100, 100 µg/ml Proteinase K, pH 8.0) for 2 h at 37 °C. Genomic DNA was extracted with phenol–chloroform and precipitated with isopropanol. Two of the samples used for DNA methylation derived from organoids cultured without Matrigel from day 0 to 77.

### RNA extraction and RNA sequencing

Total RNA from forebrain organoids or patients’ tumor biopsies was extracted using TRIzol Reagent (Invitrogen, 15596018), following manufacturer’s procedures. RNA quality was checked using the High Sensitivity RNA Assay at the 2100 Bioanalyzer (Agilent, G2939BA) and the extracted RNA was stored at − 80 °C until the RNA-sequencing (RNA-seq) analysis. RNA-seq was performed on the Novaseq 6000 instrument (Illumina, San Diego, CA) on an SP flowcell, at the Next-Generation Sequence Facility of University of Trento (CIBIO).

### Cryosectioning and immunofluorescence

Forebrain organoids were fixed with 4% PFA overnight at 4 °C, cryopreserved with 30% sucrose in distilled H_2_O overnight and embedded in Frozen Section Compound (Leica, 3801480). Organoids were sectioned at 20 µm using Thermo Scientific HM525 NX cryostat on glass slides (Thermofisher Scientific, J1800AMNZ) and subsequently prepared for immunofluorescence. First, sections were permeabilized and blocked with 3% BSA (Seqens In Vitro Diagnostic, 1005–70), 5% goat serum (Sigma, G6767) and 0.3% Triton X-100 (Sigma) in PBS 1X (permeabilization/blocking solution) for 1 h at room temperature. Primary antibodies were diluted in 3% BSA, 1% goat serum and 0.1% Triton X-100 in PBS 1X (antibody solution) and incubated at 4 °C overnight. Organoids sections were incubated with secondary antibodies (diluted in the antibody solution) for 1 h at room temperature and nuclei were counterstained with 1 µg/ml DAPI (Sigma). Mouse brains were dissected and postfixed for 24 h with 4% PFA at 4 °C. Tissues were subsequently washed with PBS and cryopreserved with 30% sucrose in distilled water and embedded in Frozen Section Compound (Leica, 3801480). Immunofluorescence was performed as described for organoids.

The antibodies used for immunofluorescence are listed below:

**Table:** 

Antibody	Host	Dilution	Company	Cat. Number
Β3-Tubulin (TUBB3)	Mouse	1:1000	Biolegend	801,201
BRAF V600E	Mouse	1:100	Abcam	Ab22846 (VE1)
Cleaved Caspase 3 (D175)	Rabbit	1:200	Cell Signaling Technology	9661S
Foxg1	Rabbit	1:200	Abcam	ab18259
GFP	Chicken	1:1000	Abcam	ab13970
Human Nuclear Antigen (HNA)	Mouse	1:200	Abcam	ab191181
Ki67	Rabbit	1:500	Abcam	ab15580
Olig2	Rabbit	1:500	Millipore	AB9610
p16	Mouse	1:200	BD Pharmingen	51-1325GR
p21	Rabbit	1:400	Abcam	ab109520
p27-KIP1	Rabbit	1:800	Cell Signaling	3686S
Satb2	Mouse	1:100	Abcam	ab51502
Sox2	Mouse	1:200	Abcam	ab171380
Tbr1	Rabbit	1:200	Abcam	ab31940
Tbr2	Rabbit	1:200	Abcam	ab23345
Alexa Fluor 488 goat anti-chicken IgG	Goat	1:500	Thermofisher Scientific	A11039
Alexa Fluor 488 goat anti-mouse IgG	Goat	1:500	Thermofisher Scientific	A11001
Alexa Fluor 546 goat anti-rabbit IgG	Goat	1:500	Thermofisher Scientific	A11035
Alexa Fluor 546 goat anti-mouse IgG	Goat	1:500	Thermofisher Scientific	A11030
Alexa Fluor 647 goat anti-rabbit IgG	Goat	1:500	Thermofisher Scientific	A21245
Alexa Fluor 647 goat anti-mouse IgG	Goat	1:500	Thermofisher Scientific	A21235

Forebrain organoids and mouse brain cryosections were imaged using Leica TCS Sp8 confocal microscope and acquired using Leica Application Suite X (LAS X) with HC PL APO 20x/0.75 CS2 objective. Mosaic acquisitions were obtained using NIS-Element on Nikon Eclipse Ti2 equipped with CREST Optics X-Light V2 Spinning Disk. Images were processed using Fiji software.

### Histology and immunohistochemistry

Formalin-Fixed Paraffin-Embedded (FFPE) 3- µm-thick sections from mice samples were cut and stained with haematoxylin and eosin (H&E) with a pre-step of deparaffination with xylol. An immunohistochemical panel including antibodies for Glial Fibrillary Acid Protein (GFAP) (Novocastra – Leica Biosystems clone GA5), Synaptophysin (Leica – clone 27G12), Ki67 (Spring – clone SP6), p16 (BD BIOSCIENCE – clone 550834) and BRAF V600E (Abcam – clone ab228461) was performed. Immunohistochemistry (IHC) was performed by Leica Bond RXm™ automated staining processor (Leica Biosystems, Buffalo Grove, IL, USA). Tissue sections were dried at 70 ºC for 30 min and then dewaxed. Antigen retrieval was performed in the Bond Rx system with Epitope Retrieval Solution 1 (pH 6) for 30 min. Sections were incubated for 30 min with the antibodies.

### Senescence-associated β-galactosidase (sGAL) staining

Organoids’ cryosections were left to thaw at room temperature for 20–30 min and subsequently washed in PBS (3 × 10 min). After the washes, they were incubated in 500 μL of X-Gal staining solution (40 mM citric acid phosphate buffer, 5 mM potassium hexanocyanoferrate(II) trihydrate, 5 mM potassium hexanocyanoferrate(III), 150 mM NaCl, 2 mM MgCl_2_−6H_2_O, 2.44 mM x-Gal) for 2 h at 37 °C. Slides were then washed in PBS (3 × 10 min) and mounted using permanent mounting media (aqueous, Histo-Line Laboratories). Slides were then stored at + 4 °C for further analysis. Images were acquired with a Zeiss Axio Imager M2 (Axiocam MRc) and processed using Fiji software.

### Immunofluorescence of FFPE sections

Immunofluorescence staining was conducted on rehydrated paraffin sections from the 15 samples reported in Figure S3f. Antigen retrieval was achieved by incubating the tissue sections in a retrieval solution composed of 10 mM sodium citrate and 0.5% Tween 20 (v/v) at pH 6.0 for 30 min at 98 °C. Blocking and antibody incubation steps were performed in PBS supplemented with 3% goat serum and 0.3% Triton X-100 (Sigma-Aldrich). For the detection of Venus^+^ cells, a polyclonal anti-GFP antibody (see table in “ Cryosectioning and immunofluorescence” section) was diluted 1:750 in antibody solution and incubated overnight at 4 °C. Secondary labelling was performed using Alexa Fluor 488-conjugated goat anti-chicken IgY (A11039), diluted 1:500 in the same antibody solution, followed by a 1-h incubation at room temperature. Nuclear counterstaining was performed with 1 μg/ml DAPI(Sigma). Stained sections were finally mounted with a permanent mounting medium to preserve fluorescence. Microscopic evaluation was initiated at 20 × magnification using an Axio Imager M1 fluorescence microscope (Carl Zeiss), aligning the fluorescence patterns with the corresponding H&E-stained sections. GFP-positive cells were subsequently analyzed under 100 × magnification, with approximately 15–20 cells examined per field. Images were acquired using ISIS image analysis software (MetaSystems). For optimal visualization, sequential filters for DAPI (blue) and FITC (green) were employed, capturing five focal planes (spaced 0.5 µm apart) for each section. Immunohistochemical staining for GFAP, Synaptophysin, BRAF V600E and p16 as well as the immunofluorescence findings were assessed using the following standardized parameters:

- Presence of tumor cells: (-) absent; (+) rare; (++) present; (+++) abundant.
- Parenchymal infiltration: (-) absent; (+) rare; (++) present; (+++) abundant.
- Vascular invasion: (-) absent; (+) rare; (++) present; (+++) abundant.

This standardized evaluation approach facilitated the consistent and reproducible assessment. The Ki67 value has been provided by scoring the counting of at least 500 neoplastic cells. The Ki67 index was expressed as the percentage of positive stained cells among the total neoplastic cells in the scored area.

### DNA methylation profiling and analysis

DNA methylation profiling was performed as previously described (PMID: 38,637,626) [22] using the Infinium Methylation EPIC (850 k) BeadChip v1 and v2 (Illumina, San Diego, CA, USA) following the manufacturer’s protocol. Methylation class assignment was conducted using version 12.5/12.8 of the DKFZ/Heidelberg Brain Tumor Classifier (https://epignostix.com/) [31]. Raw methylation data were processed in R (v4.3.1) and compared with two published DNA methylation datasets using t-Distributed Stochastic Neighbor Embedding (t-SNE) visualization.

The first dataset included tumor tissues representing diverse central nervous system (CNS) histologies [21], including reference samples from Capper et al. 2018 [31]. This dataset was chosen to assess the similarity of the study samples’ DNA methylation profiles to known neoplastic entities. The second dataset specifically consisted of pediatric brain tumors (pBT) and pBT-derived cell cultures (adapted from Pedace et al., 2024) [22]. In this dataset, cellular models that did not faithfully recapitulate their tissues of origin formed a distinct cluster in the t-SNE, suggesting a shared epigenetic drift. Study samples (n = 1 tumor sample deriving from an immunodeficient mouse grafted with *BRAF*^*V600E*^-overexpressing organoids; n = 3 tumor samples deriving from three immunodeficient mice grafted with *KIAA1549::BRAF*-overexpressing organoids) were compared to this dataset to verify their relationship with previously characterized faithful and unfaithful cell models. For reproducibility, the analysis adhered to the methodologies described by the original authors of each dataset, and thus were performed separately.

For both datasets, data loading and probe filtering were conducted using the ChAMP package [32]. Probes on SNPs, sex chromosomes, or with a detection p-value > 0.01 were excluded. Arrays from different platforms (HumanMethylation450, EPIC, or EPICv2) were processed using the "minfi" package [33], and raw beta values were merged and normalized with the BMIQ method [34].

For the first dataset, batch effects were corrected using the removeBatchEffect function from the *limma* package. The 10,000 probes with the highest standard deviation were used to calculate the 1-variance weighted Pearson correlation matrix, which was converted to a distance matrix and used as input for the Rtsne function from the *Rtsne* package (https://CRAN.R-project.org/package=Rtsne).

The second dataset consisted of 232 reference samples (149 brain tumors and 83 cell models), including 223 previously characterized by Pedace et al. 2024, along with six additional cell models and three brain tumor tissues. For this analysis, we employed the *snifter* (v1.4.0) R package (https://rdrr.io/bioc/snifter/) for PCA initialization (20 components), followed by t-SNE with hyperparameters: θ = 0.5, e.e.i. = 200, and perplexity = 15, as reported by Pedace et al. 2024.

We also derived copy number variation profiles from the raw DNA methylation data of each study sample, using R package conumee2.

### Transcriptome profiling and analysis

Transcriptome profiling was conducted on two organoids from the study group using a reference set of three unpublished low-grade tumor samples, four high-grade tumor tissues, and 12 high-grade cell models previously described in the literature [21]. RNA-seq reads were aligned to the hg38 reference genome and quantified using *Salmon* (v1.9.0). Gene counts were imported into R using the *tximeta* package (v1.14.1) and normalized with variance-stabilizing transformation. Principal component analysis (PCA) was performed on the 500 most variable genes using the plotPCA function from the *DESeq2* package. We used the same package to perform differential gene expression analysis, comparing high vs. low grade tissues and organoids separately. We characterized the common differentially expressed genes between the two comparisons performing ReactomeDB pathway enrichment analysis through WebGestalt online platform (https://www.webgestalt.org/) and selecting items related to cell senescence and MAP kinase pathway.

### Drug treatment

For drug treatment experiments, hiPSC-derived dorsal forebrain organoids electroporated with *KIAA1549::BRAF*, *BRAF*^*V600E*^ at day 77 + 300 or 77 + 320 of differentiation were cultured in Ibidi uncoated 96‐well black μ‐plates (Ibidi, 89621), placed in a 37 °C, 5% CO_2_ incubator. Organoids were treated for 7 days in CDM4 medium added with 1 μM tovorafenib (Selleckchem, S7121) or DMSO (vehicle). A complete change medium was performed every 48 h. Organoids were collected and either snap frozen in liquid nitrogen and stored at − 80 °C for western blot analysis or fixed in 4% PFA overnight at 4 °C, and stained as previously described (see “ Cryosectioning and immunofluorescence” section).

A375 melanoma cells were seeded at a density of 5,000 cells per well in 96-well plates (Corning, 3596) and allowed to adhere overnight. The following day, cells were treated with tovorafenib (0.5, 1, 5, 10, 15, 20, and 25 μM), with simultaneous addition of CellEvent™ Caspase-3/7 Green ReadyProbes™ Reagent (Thermo Fisher Scientific) at a final dilution of 1:100. Cells were monitored using the IncuCyte S3 Live-Cell Analysis System (Sartorius), acquiring five images per well every 4 h in both phase-contrast and green fluorescence channels (300 ms exposure). Cell growth was quantified as phase area confluence normalized to treatment initiation (t = 0 h), while apoptosis was assessed as the number of Caspase-3/7–positive (green) objects per image. Dose–response analysis at 3 days was performed using a four-parameter logistic nonlinear regression model to calculate the half-maximal effective concentration (EC50) for apoptosis induction.

### Quantification and statistical analysis

The quantification for Ki67^+^/Venus^+^, OLIG2^+^/Venus^+^, p21^+^/Venus^+^ and p27^+^/Venus^+^ cells was performed using macros on Fiji software. Briefly, for each image the maximum intensity projection was executed, and each channel was separated and appropriately labeled. Then, the macro segmented each image based on DAPI staining, to obtain the region of interest (ROI) of each nucleus. Successively, the macro calculated the area and mean intensity for each channel taken into consideration. The resulting data were filtered for the ROIs with Venus mean intensity higher than a set threshold. The ratio was performed by counting the Venus^+^ ROIs with mean intensity for the marker of interest higher than a set threshold, divided by the number of all counted Venus^+^ cells. Each organoid represents a point in the graph. Lastly, data were compared using a Kruskal–Wallis non-parametric statistical test with Dunn’s post hoc correction, after assessing the non-normal distribution of the data. Alternatively, data derived from four different groups were compared using Brown-Frosythe and Welch ANOVA tests.

The quantification of Cleaved Caspase 3 was performed using macros on Fiji software. Briefly, for each image the maximum intensity projection was executed, and each channel was separated and appropriately labeled. Then, the macro segmented each image based on DAPI staining, to obtain the nuclei regions of interest (nucleus ROI) of each nucleus. Each nucleus ROI was enlarged by a set number of pixel (3 pixels) to comprehend the cytosolic region (Enlarged ROI). Successively, the macro calculated the area, integrated density and mean intensity for each channel taken into consideration. The resulting data were filtered for the nucleus ROIs with Venus mean intensity higher than a set threshold. The cytosolic Cleaved Caspase 3 mean intensity was calculated as the difference between enlarged integrated density and the nuclear integrated density, divided by the difference between the enlarged ROI area and the nuclear ROI area. The ratio was performed by counting the Venus^+^ ROIs with cytosolic mean intensity for the marker of interest higher than a set threshold, divided by the number of all counted Venus + cells. Each organoid represents a point in the graph. The Shapiro–Wilk test was used to validate the assumption of normality. Statistical significance was determined using either the Mann–Whitney test for data with non‐normal distribution or the parametric two-tailed T-test with Welch’s correction for data with normal distribution.

All statistical tests and sample sizes are included in the Figure Legends and text. Statistical tests were performed with GraphPad Prism 9 software.

### Western blot analysis and quantification

Cells were lysed in Tris–HCl pH 7.6, 50 mM, deoxycholic acid sodium salt 0.5%, NaCl 140 mM, NP40 1%, EDTA 5 mM, NaF 100 mM, Na pyrophosphate 2 mM and protease inhibitors. Protein lysates were separated on 10% acrylamide gel and immunoblotted using standard procedures. The following primary antibodies were used: anti ERK 1/2 (9102S, Cell signaling), phospho-ERK1/2 (Thr202/Tyr204) (E10) (9106S, Cell signaling).

HRP-conjugated secondary antibodies (Thermofisher Scientific) were used in combination with enhanced chemiluminescence (Immobilon Western Chemiluminescent HRP Substrate, Millipore) and images of the membranes were acquired with iBright Imaging Systems (Thermo Fisher Scientific). The quantification of immunoreactivity corresponding to the bands was performed by densitometric analysis using Fiji software.

Quantitative data were presented as mean ± SEM. Prior to statistical significance testing, data were tested for normal distribution. All statistical analyses were performed with GraphPad Prism 9 software.

### scRNA-seq analysis

Raw sequencing data for each of the four organoid samples were processed using the 10 × Genomics Cell Ranger pipeline (v. 9.0.1). *BRAF*^*V600E*^ and *KIAA1549::BRAF*-expressing organoids at D77 + 70 (one organoid per sample) and at D77 + 214 (three organoids per sample) timepoints were analyzed. The cellranger count command was used to align reads to the human reference transcriptome (GRCh38) and generate single-cell gene expression matrices. The library chemistry was specified as 'Single Cell 3' v4 (–chemistry = SC3Pv4). The pipeline was executed using 16 CPU cores and 128 GB of memory, and a position-sorted BAM file was generated for each sample. The resulting filtered feature-barcode matrices from each run were used for all downstream analyses.

In particular, sequent data analysis was carried out using R 4.5.0 (https://www.R-project.org/) and Seurat v. 5.3.0. The matrices were loaded using load10x() function and Seurat objects were created using CreateSeuratObject() with min.cells parameter set to 1. The objects were merged using Merge_Seurat_List(). Quality control analyses were performed, in particular the percentage of mitochondrial RNA was calculated using PercentageFeatureSet() function on the object (pattern = "^MT", assay = "RNA"). Cells were retained for downstream analysis if they met the following criteria: expressed more than 350 and fewer than 1,250 unique features (nFeature_RNA), had a total UMI count (nCount_RNA) below 2,000 to remove potential doublets, and exhibited a mitochondrial gene content of less than 15%.

The number of cells obtained are reported in the following table.:

**Table:** 

Sample	Cells before filtering	Cells after filtering
KIAA1549::BRAF D77 + 70	662	456
KIAA1549::BRAF D77 + 214	2318	2027
BRAF^V600E^ D77 + 70	2554	1356
BRAF^V600E^ D77 + 214	3557	2245

This filtered organoid object was then processed using the standard Seurat workflow. Data was log-normalized (NormalizeData), and 5,000 highly variable features were identified (FindVariableFeatures), with the *BRAF* gene explicitly added to this list. The data was then scaled (ScaleData), and principal component analysis (PCA) was performed (RunPCA).

To compare organoid-derived cells with primary tumor cells, a publicly available single-cell RNA-seq dataset from pilocytic astrocytoma patients was acquired (Reitman et al., *Nature Communications*, 2019; SCP271) [24]. The raw count matrix and corresponding metadata were requested and kindly sent by the authors and used to create a Seurat object. To ensure a fair comparison with the organoid model, which lacks an immune compartment, cells from the Reitman dataset annotated as immune cluster were excluded by subsetting the object to retain only cells within the "tumor cluster" as defined by the original authors (“Supercluster” metadata column). This filtered patient object was then processed independently using the same Seurat workflow as the organoid data: normalization, identification of 5,000 variable features (including *BRAF*), scaling, and PCA.

The processed organoid object and the processed, tumor-only patient object were combined into a single Seurat object using the Merge_Seurat_List function. To correct for batch effects between the two datasets, we used the JointPCA integration method (method = JointPCAIntegration) as implemented in Seurat v5. The integration was performed starting from the original PCA calculated on the merged object (orig.reduction = "pca"), and the resulting corrected dimensionality reduction was named integrated.jointpca.

Following integration, downstream analysis was performed on this corrected reduction. A shared nearest neighbor (SNN) graph was constructed using FindNeighbors based on the first 30 dimensions (dims = 1:30) of the integrated.jointpca reduction. Cell clusters were then identified using FindClusters with a resolution of 0.5. Finally, a new UMAP embedding for visualization, named umap.integrated.jointpca, was calculated using the first 30 dimensions of the integrated JointPCA reduction. This final, integrated object was used for all subsequent annotation and comparative analyses.

The Supplementary Data 6 table of Gene Programs identified in pilocytic astrocytoma tumors cells was downloaded from Reitman et al., *Nature Communications*, 2019. A column was added, consisting of the union of the MAPK signaling and AC-like gene programs, and it was named MAPK signaling/AC-like gene program. Using pivot_longer() from tidyr v. 1.3.1 the table was transformed in a longer format, required from the cellmarkeraccordion package. For subsequent analyses cellmarkeraccordion v 1.0.0 [25] was used to annotate the dataset, in particular the function accordion_custom() was used, with min_n_marker set to 3, allow_unknown set to FALSE and annotation_resolution set to cluster.

For the cell type annotation, accordion_disease() was used, selecting “low grade glioma” and “high grade glioma” as disease. For both executions of accordion_custom() and accordion_disease() other parameters that were set are: min_n_markers = 3, annotation_resolution = "cluster", plot = TRUE. Other parameters were default.

UMAP plots were created using FeaturePlot() and DimPlot() functions, using ggplot2 v. 3.5.2 for better visualization. The same package was used to create the barplots.

Top Markers and Competing Annotation plots were retrieved from the misc slot of the Seurat object, which were generated using the parameter plot = TRUE of the accordion_custom() function.

To calculate the markers of each cluster and of cells classified as AC/OC-like vs MAPK signaling gene programs, findAllMarkers function of Seurat was employed, using Wilcoxon test with default parameters (logfc.threshold = 0.1, slot = "data", min.pct = 0.01, min.diff.pct = -Inf, node = NULL, only.pos = FALSE,max.cells.per.ident = Inf,random.seed = 1,latent.vars = NULL, min.cells.feature = 3, min.cells.group = 3, mean.fxn = NULL,fc.name = NULL, base = 2, return.thresh = 0.01, densify = FALSE).

Then to perform enrichment analyses the library clusterprofiler (v. 4.16.0) was employed, in particular the function compareCluster(), using the Benjamini Hochberg method, the positive significant markers, and the genes present in the object as the universe. In particular for the Gene Ontology enrichment analysis the following parameter were used: fun = "enrichGO", OrgDb = "org.Hs.eg.db", ont = "BP", pvalueCutoff = 0.05, qvalueCutoff = 0.05. Similarly, for the KEGG enrichment analysis the parameters were: fun = "enrichKEGG", organism = "hsa", pvalueCutoff = 0.05. For the visualization, the function dotplot of the library enrichplot (v. 1.28.4, ttps://bioconductor.org/packages/enrichplot) was employed.

## Supplementary Information

Supplementary Material 1: Supplementary Figure 1. Generation of human pLGG organoid models. a, b) Schematic representation of the protocol (a) and of the genetic combinations (b) used for organoids electroporation at day 35 of differentiation. c) Live imaging of forebrain organoids electroporated at day 35 of differentiation with Venus, *BRAF*^*V600E*^, *KIAA1549::BRAF*, or *TPR-MET+p53*^R273C^. Organoids were imaged at 15 (D35+15), 30 (D35+30), 45 (D35+45) and 70 (D35+70) days post electroporation. Supplementary Figure 2. *BRAF*^V600E^ and *KIAA1549::BRAF* overexpression at day 35 of differentiation. a,b) Confocal images of immunofluorescence of Venus and Ki67 (a) or OLIG2 (b) in hiPSC-derived dorsal forebrain organoids electroporated with Venus, *BRAF*^V600E^, *KIAA1549::BRAF*, *TPR-MET+p53*^R273C^ at day 35+30. c,d) Quantifications of Ki67^+^ (c) or OLIG2^+^ (d) cells co-expressing Venus in hiPSC-derived dorsal forebrain organoids at day 35+30 of differentiation electroporated with Venus, *BRAF*^V600E^,*KIAA1549::BRAF*, *TPR-MET+p53*^R273C^. Data are presented as mean ± S.E.M.; each dot represents the signal coming from a single organoid. For each marker, n = 2–5 images were considered. Statistics: Kruskal–Wallis test with Dunn’s post hoc correction;*p ≤ 0.05; **p ≤ 0.01; ***p ≤ 0.001. Scale (a, b) 100µm. Supplementary Figure 3. *In vivo* grafting of human pLGG organoids mimic patient tumors. a, b) Representative images of immunofluorescence of *BRAF*^V600E^ and Venus-expressing cells in hiPSC-derived dorsal forebrain organoids electroporated with *TPR-MET+p53*^R273C^(a) and *BRAF*^V600E^ (b). at day 77+70. c, d) Representative images of Ki67-expressing cells in brain cryosections of immunodeficient mice orthotopically engrafted with (c) *BRAF*^V600E^- and (d) *KIAA1549::BRAF*-overexpressing organoids. e) Representative images of p16-expressing cells in brain cryosections of immunodeficient mice orthotopically engrafted with *BRAF*^V600E^- and *KIAA1549::BRAF*-overexpressing organoids. f) Summary table of the histological evaluation of mouse brains engrafted with organoids at D77+70 of differentiation and electroporated with *BRAF*^V600E^ or *KIAA1549::BRAF*. Analysis of samples based on *BRAF* gene status. Descriptive summary of tumor presence, histology, parenchymal and vascular infiltration, and immunohistochemical evaluation. Each ID represents a mouse (identified by the letters) engrafted with pLGG organoids (identified by numbers, two biological replicates per combination). (ID: identification number; LGG: low grade glioma; HGG: high grade glioma; moderate: moderately cellular tumor with no evidence of high-grade features: NA: not analysed). low grade with Ki67 < 3%, moderate >3 and <10, high-grade >10. g) Mosaic image of Human Nuclear Antigen (HNA)-expressing cells in brain cryosections of immunodeficient mice orthotopically engrafted with wildtype forebrain organoids at day 70 of differentiation. h) Representative image of Ki67 and Human Nuclear Antigen (HNA)-expressing cells in brain cryosections of immunodeficient mice engrafted with wildtype forebrain organoids at day 70 of differentiation. i) Mosaic image of mouse brain engrafted with forebrain organoids electroporated with *TPR-MET+p53*^R273C^ at day 35+30 of differentiation. Clusters of mCherry+ cells are highlighted in the insets (i’,i’’). i’,i’’) Representative images of p16, mCherry, p21 and Human Nuclear Antigen (HNA)-expressing cells in brain cryosections of immunodeficient mice engrafted with *TPR-MET+p53*^R273C^ at day 35+30 of differentiation. Scale bars (a,b) 10µm, (c-e) 100µm, (g,i) 5mm, (h) 50µm. Supplementary Figure 4. Methylation-based copy number variation plots of pLGG organoids and differential gene expression analysis from bulk transcriptome. a-d) Blue lines represent the normalized copy number across each segment. Dark red dots represent gains, while blue dots represent losses. Panels a, b, c, d represent samples Org1, Org2, Org3 and Org4 respectively. e) Venn diagram summarizing the results of the differential gene expression analysis. f) ReactomeDB pathway enrichment analysis of the common differentially expressed genes between low- vs. high-grade tissues and cell models. Enriched pathways of interest are represented on the Y-axis. For each one, the X-axis value represents the fold enrichment. Circle color indicates the FDR-adjusted p-value of the enrichment, while circle size varies according to the number of genes included in the pathway. Supplementary Figure 5. Characterization of late timepoint pLGG organoids. a,b) Live imaging of hiPSC-derived dorsal forebrain organoids electroporated with *KIAA1549::BRAF* at day 77+300 (a) or *BRAF*^V600E^ at day 77+320 (b) using an ImageXpress Micro Confocal High Content Imaging System (Molecular Devices). Scale bar 500 µm. c,d) Quantifications of cells expressing Venus/DAPI in hiPSC-derived dorsal forebrain organoids at day 77+30, day 77+70, and day 77+300 of differentiation electroporated with *KIAA1549::BRAF* (c) or *BRAF*^V600E^ (d). e-h) Quantifications of KI67^+^ (e), OLIG2^+^ (f), p21^+^ (g), p27^+^ (h) cells co-expressing Venus in hiPSC-derived dorsal forebrain organoids at day 77+170 of differentiation electroporated with Venus, *BRAF*^V600E^, *KIAA1549::BRAF orTPR-MET+p53*^R273C^. Data are presented as mean ± S.E.M.; each dot represents a single organoid. For each marker, n = 2–5 images were considered. Statistics: Brown-Forsythe and Welch ANOVA test; *p ≤ 0.05; **p ≤ 0.01; ***p ≤ 0.001. Supplementary Figure 6. pLGG organoid cells align with OC-like, AC-like and MAPK signature clusters. a) Barplot of *BRAF* positive cells per sample. Each bar represents the percentage of cells present in each sample colored by positivity (i.e. normalized expression > 0, in red) or negativity (blue) to *BRAF*. (b-e) UMAP visualizations of scRNA-seq data where each point represents a single cell. Colors indicate the clusters found by Seurat (see Methods, “scRNA-seq analysis” section) (b), the sample type (pLGG organoid or patient-derived tumors), (c), the sample type and timepoint of pLGG organoids (*BRAF*^*V600E*^ and *KIAA1549::BRAF*-expressing organoids at D77+70, n = 1 organoid per sample type, and at D77+214, n = 3 organoids per sample type) (d) or just the timepoint of the pLGG organoids (e). f) Barplot indicating the number of cells annotated for each gene program using markers from Reitman et al., 2019. Colors indicate the timepoint of the organoids or if the cell comes from patient-derived tumors. g) Barplot indicating the number of cells assigned to cell types using high and low grade glioma markers from cellMarkerAccordion package (see Methods, “scRNA-seq analysis” section). Colors indicate the timepoint of the organoids or if the cell comes from patient-derived tumors. h) UMAP visualization of scRNA-seq data where each point represents a single cell. Colors indicate the sample of origin. i) Barplot indicating the number of cells assigned to cell types using high and low grade glioma markers from cellMarkerAccordion package (see methods). Colors indicate the sample of origin. j) Barplot indicating the number of cells annotated for each gene program using markers from Reitman et al., 2019. Colors indicate the sample of origin. Data from all samples were computationally integrated using the JointPCA method to correct for technical batch effects. Supplementary Figure 7. Gene Ontology (GO) and Kyoto Encyclopedia of Genes and Genomes (KEGG) enriched terms. a) Dotplot of the Gene Ontology (GO) enriched terms of positive markers for each cluster. b) Dotplot of the Kyoto Encyclopedia of Genes and Genomes (KEGG) enriched terms of positive markers for each cluster. c) Dotplot of the Gene Ontology (GO) enriched terms of positive markers for AC-like/OC-like and MAPK-signaling gene programs cells. d) Dotplot of the Kyoto Encyclopedia of Genes and Genomes (KEGG) enriched terms of positive markers for AC-like/OC-like and MAPK-signaling gene programs cells. Color indicates the Benjamini Hochberg adjusted p-value, size indicates the Gene Ratio. The number in parenthesis below the cluster name indicates the number of positive markers for that cluster. Supplementary Figure 8. Effects of tovorafenib treatment on ERK signaling and cell viability in organoids and A375 melanoma cells. a) Representative western blot for ERK and its phosphorylated form (p-ERK) in hiPSC-derived dorsal forebrain organoids at day 77+300 or 77+320 of differentiation, electroporated either with *KIAA1549::BRAF* or *BRAF*^V600E^ and treated with tovorafenib 1 µM or DMSO (vehicle). Ponceau staining was used as loading control. b) A375 melanoma cells confluency. The kinetic curves show the percentage of cell confluence normalized to the confluence measured at the time of treatment initiation (t = 0 hours). At 24 hours post-treatment, cells displayed a cytostatic effect, which progressed to dose-dependent cytotoxicity over time. c) A375 melanoma cells apoptotic activity was measured as Caspase-3/7-positive cell count. A marked increase in apoptosis was observed after 24 hours, becoming detectable primarily at the highest drug concentrations. Data are presented as mean ± SEM, n = 3 technical replicates. d) The number of Caspase-3/7–positive cells per image measured at 72 hours was plotted against drug concentration, and the EC50 for apoptosis induction was calculated using the IncuCyte analysis software. The resulting EC50 was 5.79 μM.

## Data Availability

The code used to analyze all data is available via GitHub at https://github.com/LTiberiLab/pLGG_Leva_Santomaso_2026.

## References

[1] Cohen AR. Brain Tumors in Children. N Engl J Med. 2022;386:1922–31. 10.1056/NEJMra2116344 35584157

[2] Louis DN, et al. The 2016 World Health Organization classification of tumors of the central nervous system: a summary. Acta Neuropathol. 2016;131:803–20. 10.1007/s00401-016-1545-1 27157931

[3] Jones DTW, et al. Molecular characteristics and therapeutic vulnerabilities across paediatric solid tumours. Nat Rev Cancer. 2019;19:420–38. 10.1038/s41568-019-0169-x 31300807

[4] Fangusaro J, et al. A phase II trial of selumetinib in children with recurrent optic pathway and hypothalamic low-grade glioma without NF1: a Pediatric Brain Tumor Consortium study. Neuro-Oncol. 2021;23:1777–88. 10.1093/neuonc/noab047 PMC8485450 33631016

[5] Bouffet E, et al. Efficacy and Safety of Trametinib Monotherapy or in Combination With Dabrafenib in Pediatric BRAF V600-Mutant Low-Grade Glioma. J Clin Oncol. 2023;41:664–74. 10.1200/JCO.22.01000 PMC9870224 36375115

[6] Kilburn LB, et al. The type II RAF inhibitor tovorafenib in relapsed/refractory pediatric low-grade glioma: the phase 2 FIREFLY-1 trial. Nat Med. 2024;30:207–17. 10.1038/s41591-023-02668-y PMC10803270 37978284

[7] Jacob K, et al. Genetic aberrations leading to MAPK pathway activation mediate oncogene-induced senescence in sporadic pilocytic astrocytomas. Clin Cancer Res. 2011;17:4650–60. 10.1158/1078-0432.CCR-11-0127 21610151

[8] Raabe EH, et al. BRAF activation induces transformation and then senescence in human neural stem cells: a pilocytic astrocytoma model. Clin Cancer Res. 2011;17:3590–9. 10.1158/1078-0432.CCR-10-3349 PMC4086658 21636552

[9] Yvone GM and Breunig JJ. Pediatric low-grade glioma models: advances and ongoing challenges. Front. Oncol. 2024;13:1346949. 10.3389/fonc.2023.1346949 PMC10839015 38318325

[10] Abdullah KG, et al. Establishment of patient-derived organoid models of lower-grade glioma. Neuro-Oncol. 2022;24:612–23. 10.1093/neuonc/noab273 PMC8972292 34850183

[11] Lago C, et al. Patient‐ and xenograft‐derived organoids recapitulate pediatric brain tumor features and patient treatments. EMBO Mol Med. 2023;15:e18199. 10.15252/emmm.202318199 PMC10701620 38037472

[12] Ryall S, et al. Integrated Molecular and Clinical Analysis of 1,000 Pediatric Low-Grade Gliomas. Cancer Cell. 2020;37:569-583.e5. 10.1016/j.ccell.2020.03.011 PMC7169997 32289278

[13] Anastasaki C, et al. Human induced pluripotent stem cell engineering establishes a humanized mouse platform for pediatric low-grade glioma modeling. Acta Neuropathol Commun. 2022;10:120. 10.1186/s40478-022-01428-2 PMC9392324 35986378

[14] Lassaletta A, et al. Therapeutic and prognostic implications of BRAF V600E in pediatric low-grade gliomas. J Clin Oncol. 2017;35:2934–41. 10.1200/JCO.2016.71.8726 PMC5791837 28727518

[15] Schindler G, et al. Analysis of BRAF V600E mutation in 1,320 nervous system tumors reveals high mutation frequencies in pleomorphic xanthoastrocytoma, ganglioglioma and extra-cerebellar pilocytic astrocytoma. Acta Neuropathol. 2011;121:397–405. 10.1007/s00401-011-0802-6 21274720

[16] Velasco S, et al. Individual brain organoids reproducibly form cell diversity of the human cerebral cortex. Nature. 2019;570:523–7. 10.1038/s41586-019-1289-x PMC6906116 31168097

[17] Kadoshima T, et al. Self-organization of axial polarity, inside-out layer pattern, and species-specific progenitor dynamics in human ES cell–derived neocortex. Proc Natl Acad Sci USA. 2013;110:20284–9. 10.1073/pnas.1315710110 PMC3864329 24277810

[18] Antonica F, et al. A slow-cycling/quiescent cells subpopulation is involved in glioma invasiveness. Nat Commun. 2022;13:4767. 10.1038/s41467-022-32448-0 PMC9378633 35970913

[19] Kim H, et al. Pluripotent Stem Cell-Derived Cerebral Organoids Reveal Human Oligodendrogenesis with Dorsal and Ventral Origins. Stem Cell Rep. 2019;12:890–905. 10.1016/j.stemcr.2019.04.011 PMC6524754 31091434

[20] Otero JJ, Rowitch D, Vandenberg S. OLIG2 is differentially expressed in pediatric astrocytic and in ependymal neoplasms. J Neurooncol. 2011;104:423–38. 10.1007/s11060-010-0509-x PMC3161192 21193945

[21] Auffret L, et al. A new subtype of diffuse midline glioma, H3 K27 and BRAF/FGFR1 co-altered: a clinico-radiological and histomolecular characterisation. Acta Neuropathol. 2023;147:2 (Berl). 10.1007/s00401-023-02651-4 PMC10709479 38066305

[22] Pedace L, et al. Evaluating cell culture reliability in pediatric brain tumor primary cells through DNA methylation profiling. Npj Precis Oncol. 2024;8:1–13. 10.1038/s41698-024-00578-x PMC11026496 38637626

[23] Mackay A, et al. Integrated Molecular Meta-Analysis of 1,000 Pediatric High-Grade and Diffuse Intrinsic Pontine Glioma. Cancer Cell. 2017;32:520-537.e5. 10.1016/j.ccell.2017.08.017 PMC5637314 28966033

[24] Reitman ZJ, et al. Mitogenic and progenitor gene programmes in single pilocytic astrocytoma cells. Nat Commun. 2019;10:3731. 10.1038/s41467-019-11493-2 PMC6700116 31427603

[25] Busarello E, et al. Cell Marker Accordion: interpretable single-cell and spatial omics annotation in health and disease. Nat Commun. 2025;16:5399. 10.1038/s41467-025-60900-4 PMC12234662 40623970

[26] Ballabio C, et al. Modeling medulloblastoma in vivo and with human cerebellar organoids. Nat Commun. 2020;11:583. 10.1038/s41467-019-13989-3 PMC6989674 31996670

[27] Ballabio C, et al. Notch1 switches progenitor competence in inducing medulloblastoma. Sci Adv. 2021;7:eabd2781. 10.1126/sciadv.abd2781 PMC8221631 34162555

[28] Lago C, et al. Medulloblastoma and high-grade glioma organoids for drug screening, lineage tracing, co-culture and in vivo assay. Nat Protoc. 2023;18:2143–80. 10.1038/s41596-023-00839-2 37248391

[29] Nysom K, et al. Radiographic and visual response to the type II RAF inhibitor tovorafenib in children with relapsed/refractory optic pathway glioma in the FIREFLY-1 trial. Neuro Oncol. 2025;27:1341–55. 10.1093/neuonc/noae274 PMC12187376 39700439

[30] Ioannou M, et al. Clinical experience with tovorafenib in adults with treatment-refractory high- and low-grade gliomas. Neurooncol Pract. 2025;12:1051–7. 10.1093/nop/npaf068 PMC12741819 41458920

[31] Capper D, et al. DNA methylation-based classification of central nervous system tumours. Nature. 2018;555:469–74. 10.1038/nature26000 PMC6093218 29539639

[32] Tian Y, et al. ChAMP: updated methylation analysis pipeline for Illumina BeadChips. Bioinformatics. 2017;33:3982–4. 10.1093/bioinformatics/btx513 PMC5860089 28961746

[33] Aryee MJ, et al. Minfi: a flexible and comprehensive Bioconductor package for the analysis of Infinium DNA methylation microarrays. Bioinformatics. 2014;30:1363–9. 10.1093/bioinformatics/btu049 PMC4016708 24478339

[34] Teschendorff AE, et al. A beta-mixture quantile normalization method for correcting probe design bias in Illumina Infinium 450 k DNA methylation data. Bioinformatics. 2013;29:189–96. 10.1093/bioinformatics/bts680 PMC3546795 23175756

