# Supplementary material for: Modeling pediatric low-grade glioma heterogeneity using human forebrain organoids
Source: Mol Cancer. 2026 Apr 1;25:133. doi: 10.1186/s12943-026-02612-x (PMC13192117; doi:10.1186/s12943-026-02612-x)
Supplement: Supplementary file 1 — Supplementary Material 1: Supplementary Figure 1. Generation of human pLGG organoid models. a, b) Schematic representation of the protocol (a) and of the genetic combinations (b) used for organoids electroporation at day 35 of differentiation. c) Live imaging of forebrain organoids electroporated at day 35 of differentiation with Venus, BRAFV600E, KIAA1549::BRAF, or TPR-MET+p53R273C. Organoids were imaged at 15 (D35+15), 30 (D35+30), 45 (D35+45) and 70 (D35+70) days post electroporation. Supplementary Figure 2. BRAFV600E and KIAA1549::BRAF overexpression at day 35 of differentiation. a,b) Confocal images of immunofluorescence of Venus and Ki67 (a) or OLIG2 (b) in hiPSC-derived dorsal forebrain organoids electroporated with Venus, BRAFV600E, KIAA1549::BRAF, TPR-MET+p53R273C at day 35+30. c,d) Quantifications of Ki67+ (c) or OLIG2+ (d) cells co-expressing Venus in hiPSC-derived dorsal forebrain organoids at day 35+30 of differentiation electroporated with Venus, BRAFV600E,KIAA1549::BRAF, TPR-MET+p53R273C. Data are presented as mean ± S.E.M.; each dot represents the signal coming from a single organoid. For each marker, n = 2–5 images were considered. Statistics: Kruskal–Wallis test with Dunn’s post hoc correction;*p ≤ 0.05; **p ≤ 0.01; ***p ≤ 0.001. Scale (a, b) 100µm. Supplementary Figure 3. In vivo grafting of human pLGG organoids mimic patient tumors. a, b) Representative images of immunofluorescence of BRAFV600E and Venus-expressing cells in hiPSC-derived dorsal forebrain organoids electroporated with TPR-MET+p53R273C(a) and BRAFV600E (b). at day 77+70. c, d) Representative images of Ki67-expressing cells in brain cryosections of immunodeficient mice orthotopically engrafted with (c) BRAFV600E- and (d) KIAA1549::BRAF-overexpressing organoids. e) Representative images of p16-expressing cells in brain cryosections of immunodeficient mice orthotopically engrafted with BRAFV600E- and KIAA1549::BRAF-overexpressing organoids. f) Summary table of the histological evaluat [file 12943_2026_2612_MOESM1_ESM.pdf]

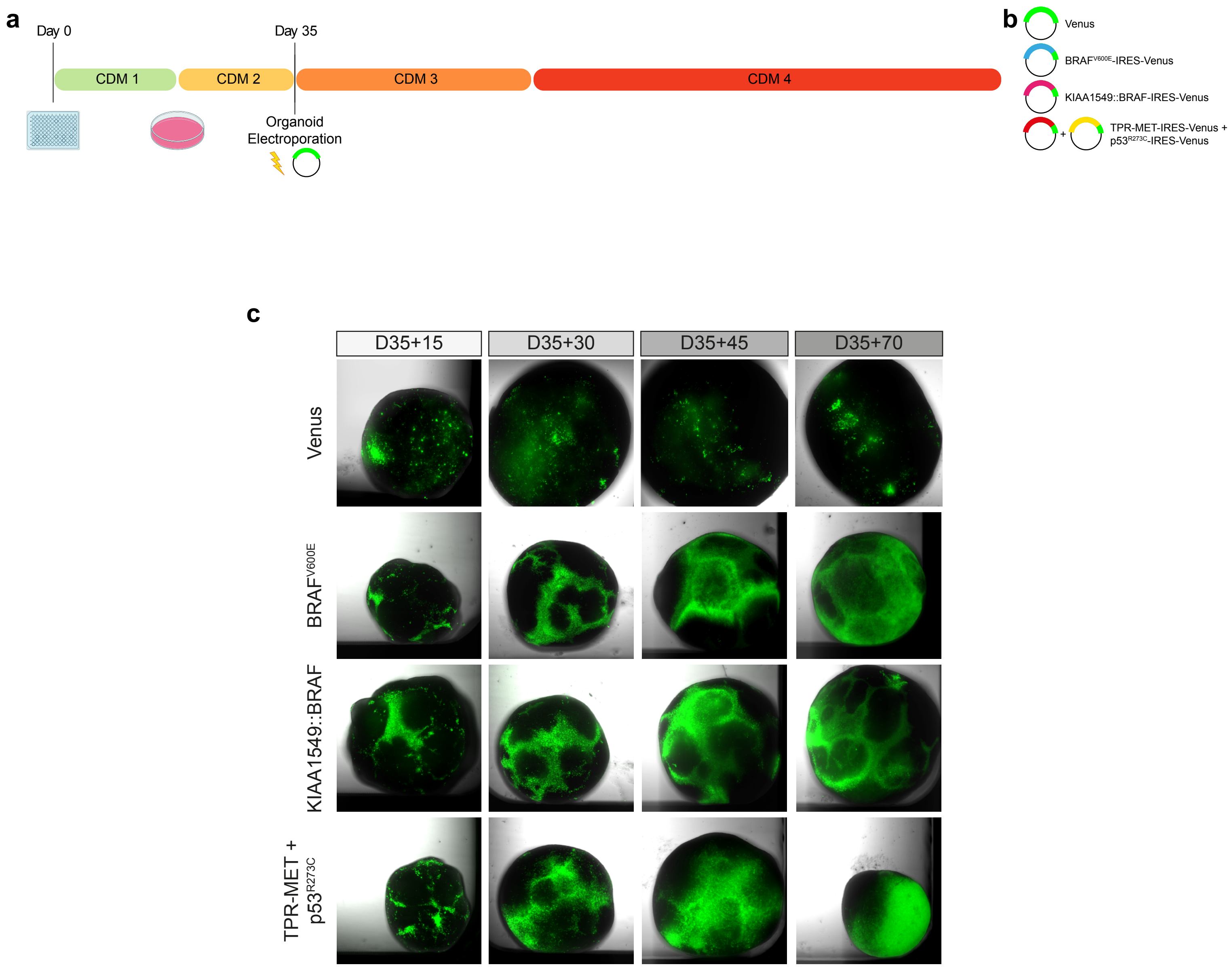

Supplementary Figure 1

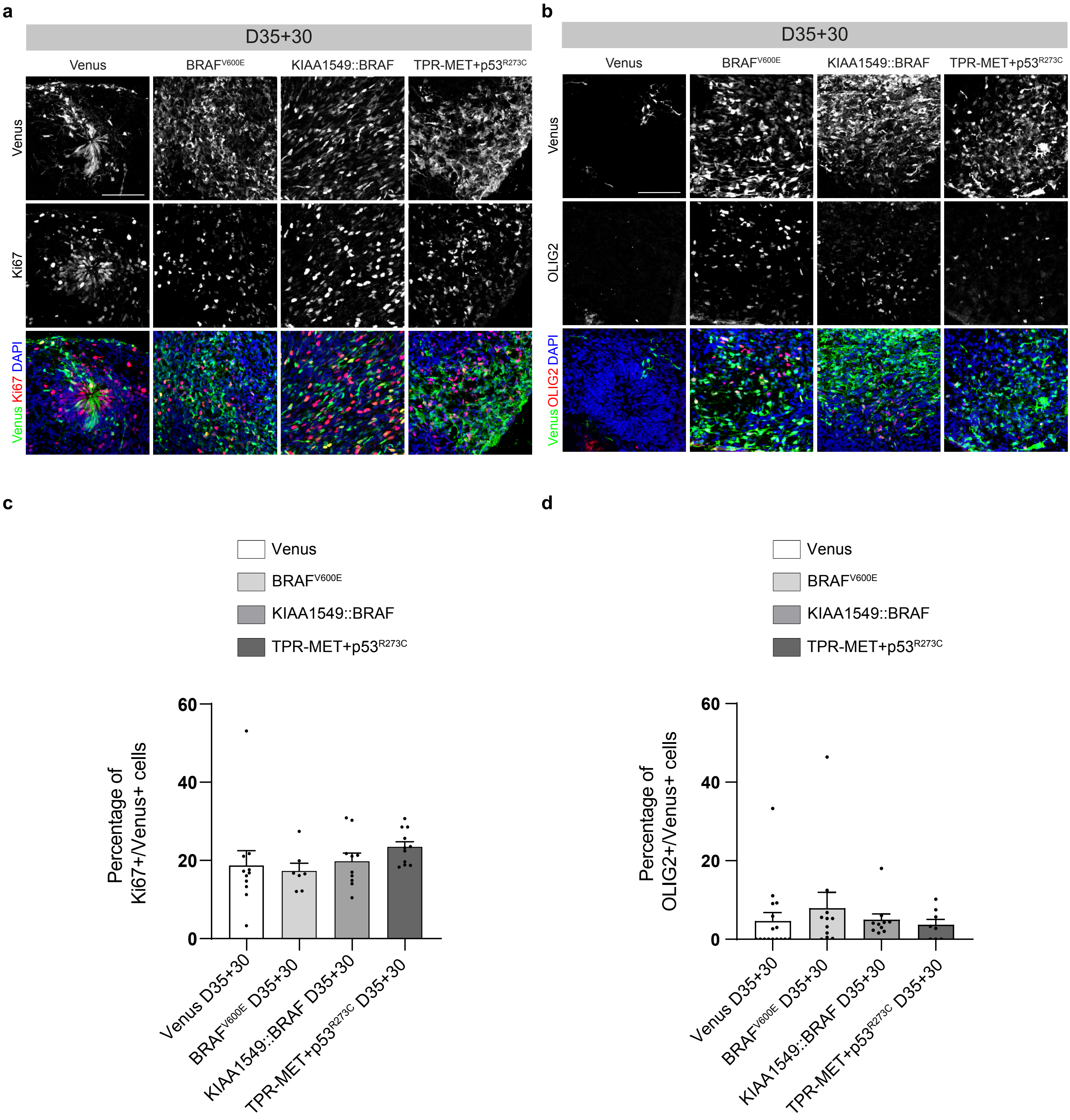

Supplementary Figure 2

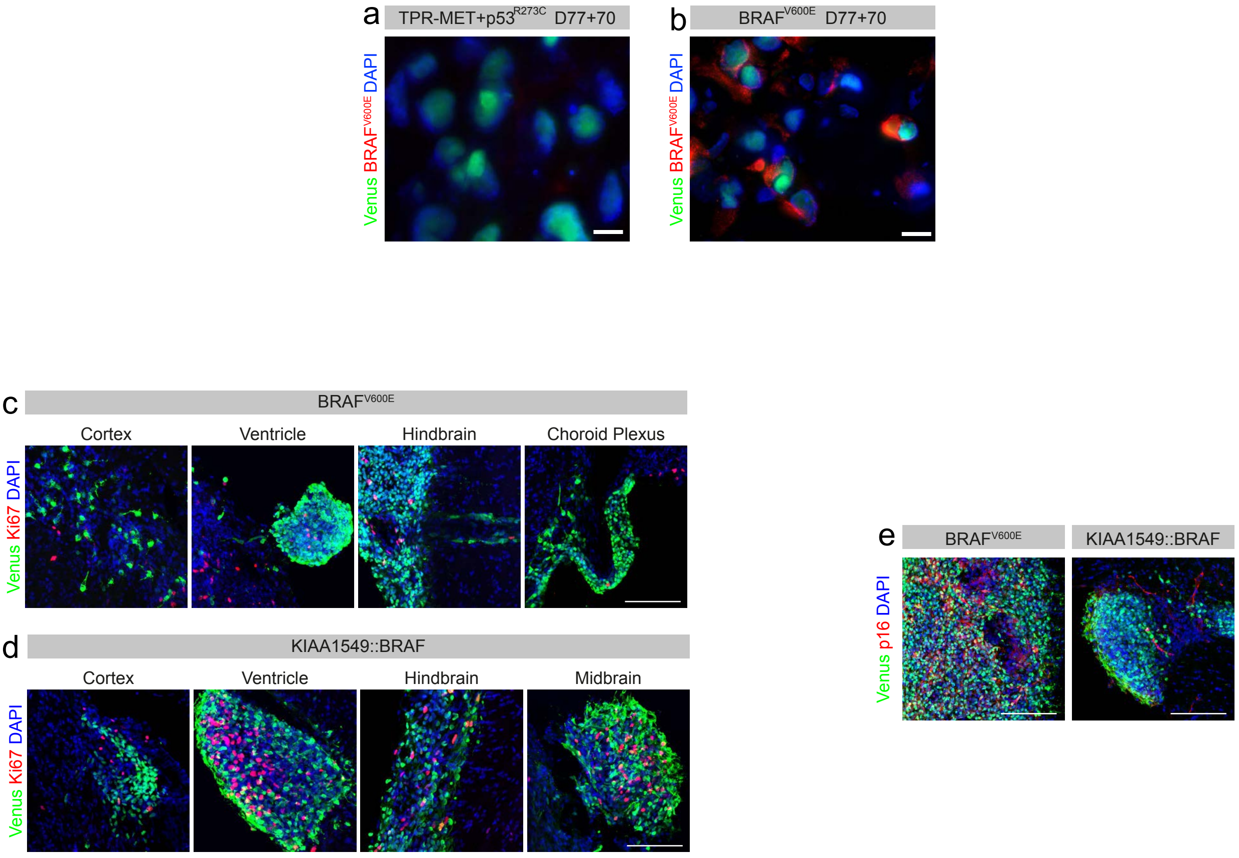

f

BRAF status	ID	Presence of tumor	Parenchymal infiltration	Vascular invasion	Histology	Immunohistochemical evaluation				
						GFAP	Synaptophysin	Ki67	BRAF^{V600E}	p16 (% of positive cells)
BRAF^{V600E}	1a	+	+	-	LGG	+		2%	+	+(30%)
	1b	+	-	-	LGG	+		2%	+	Not valuable
	1c	+	-	-	LGG	+		2%	+	+(5%)
	1d	+	-	-	LGG	+		1%	+	+(25%)
	2a	++	++	+	Moderate	+		4%	+	+(10%)
	2b	+	+	-	LGG	+	-/+	2%	+	+(30%)
	2c	+	-	-	LGG	+		2%	+	+(3%)
KIAA1549::BRAF	3a	+	-	-	HGG	-/+	+/-	85%	NA	+(90%)
	3b	+	-	-	LGG	-/+		Not valuable	NA	Not valuable
	3c	-	-	-	-	+		Not valuable	NA	Not valuable
	3d	+	-	-	LGG	+		1%	NA	Not valuable
	4a	+	+	+	HGG	++		10%	NA	+(10%)
	4b	+	-	-	LGG	+		1%	NA	+(3%)
	4c	+	+	-	LGG	+		3%	NA	Not valuable
	4d	+	+	+	Moderate	+		8%	NA	+(20%)

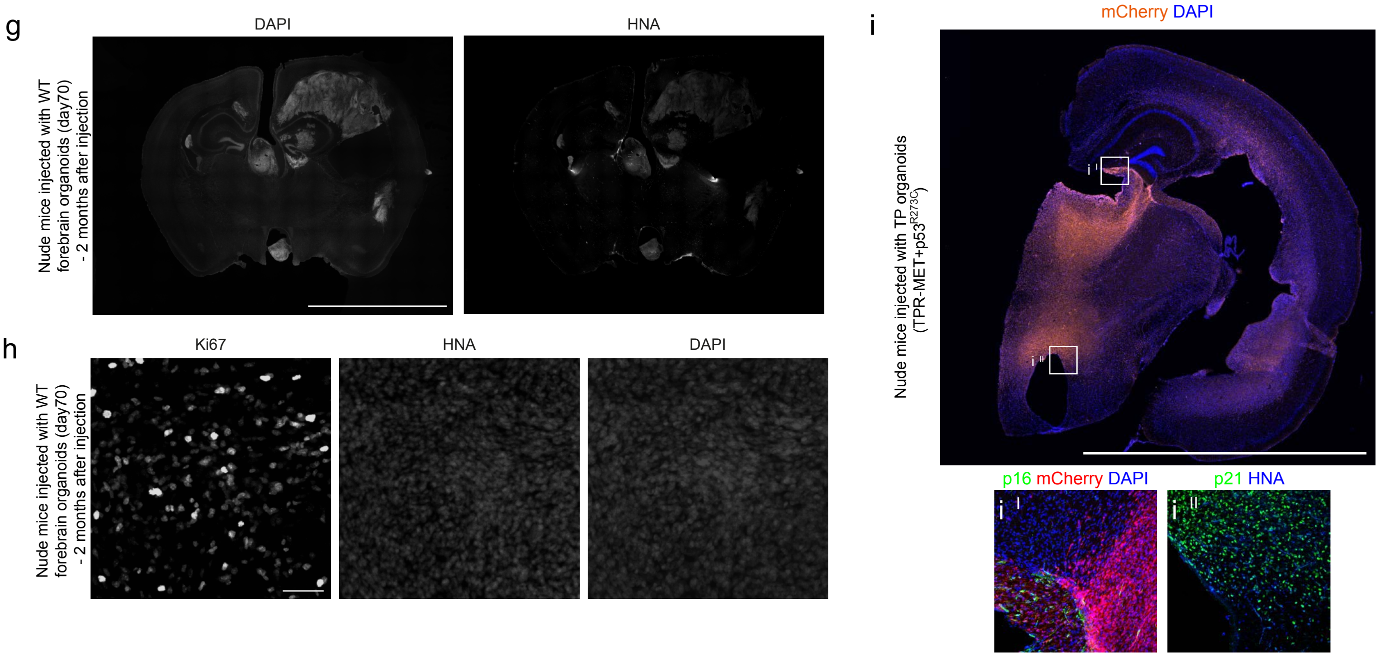

Supplementary Figure 3

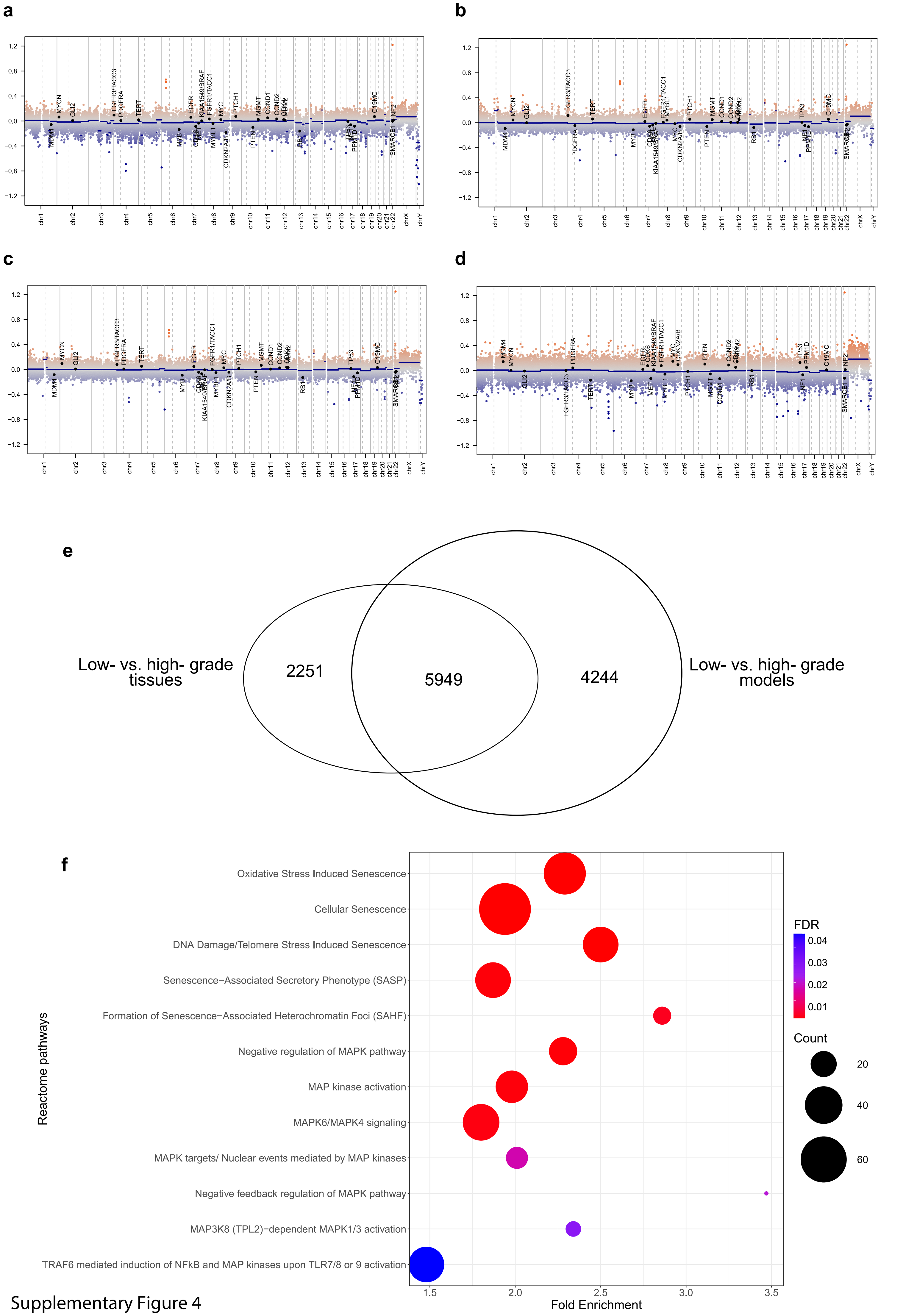

Supplementary Figure 4

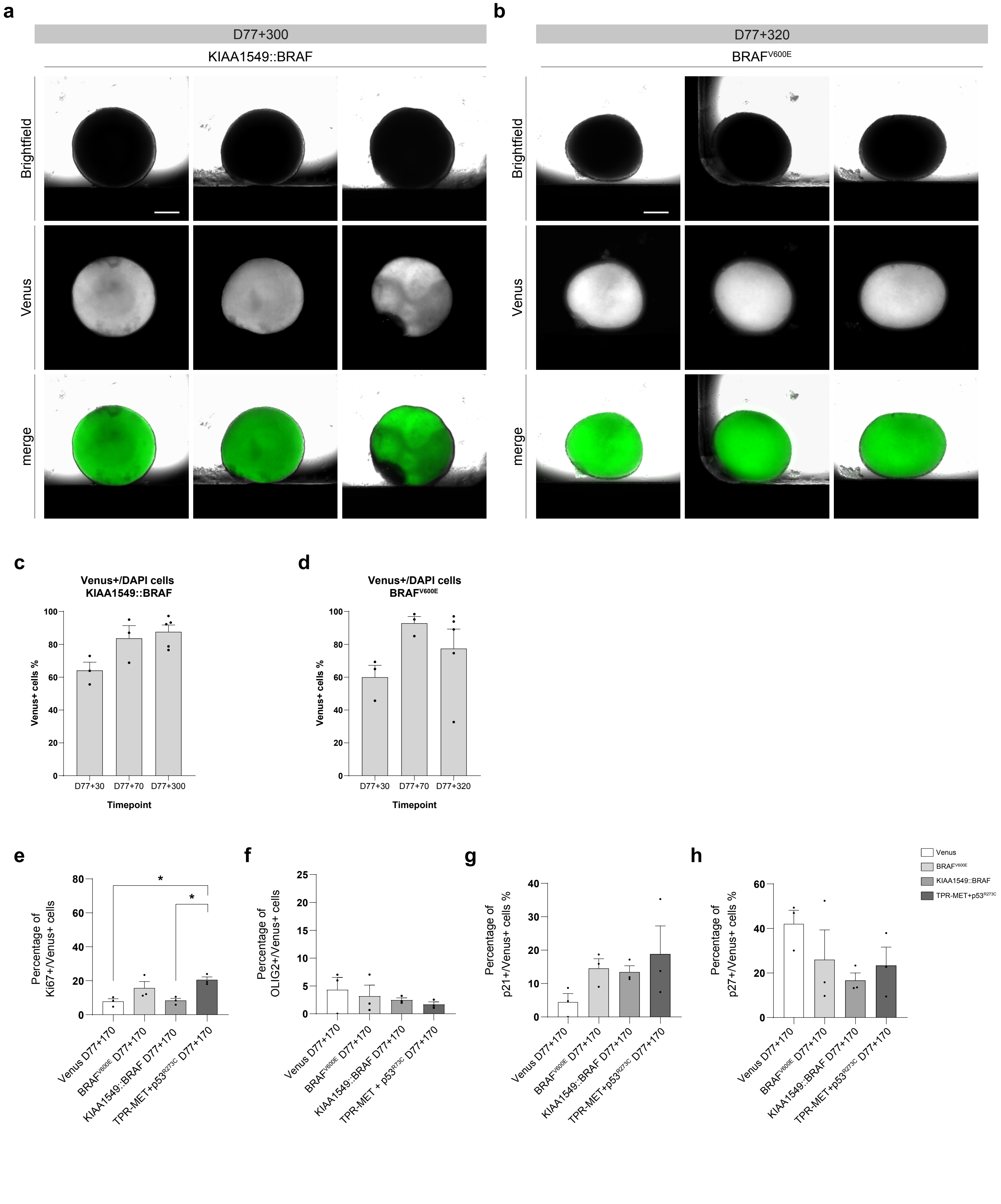

Supplementary Figure 5

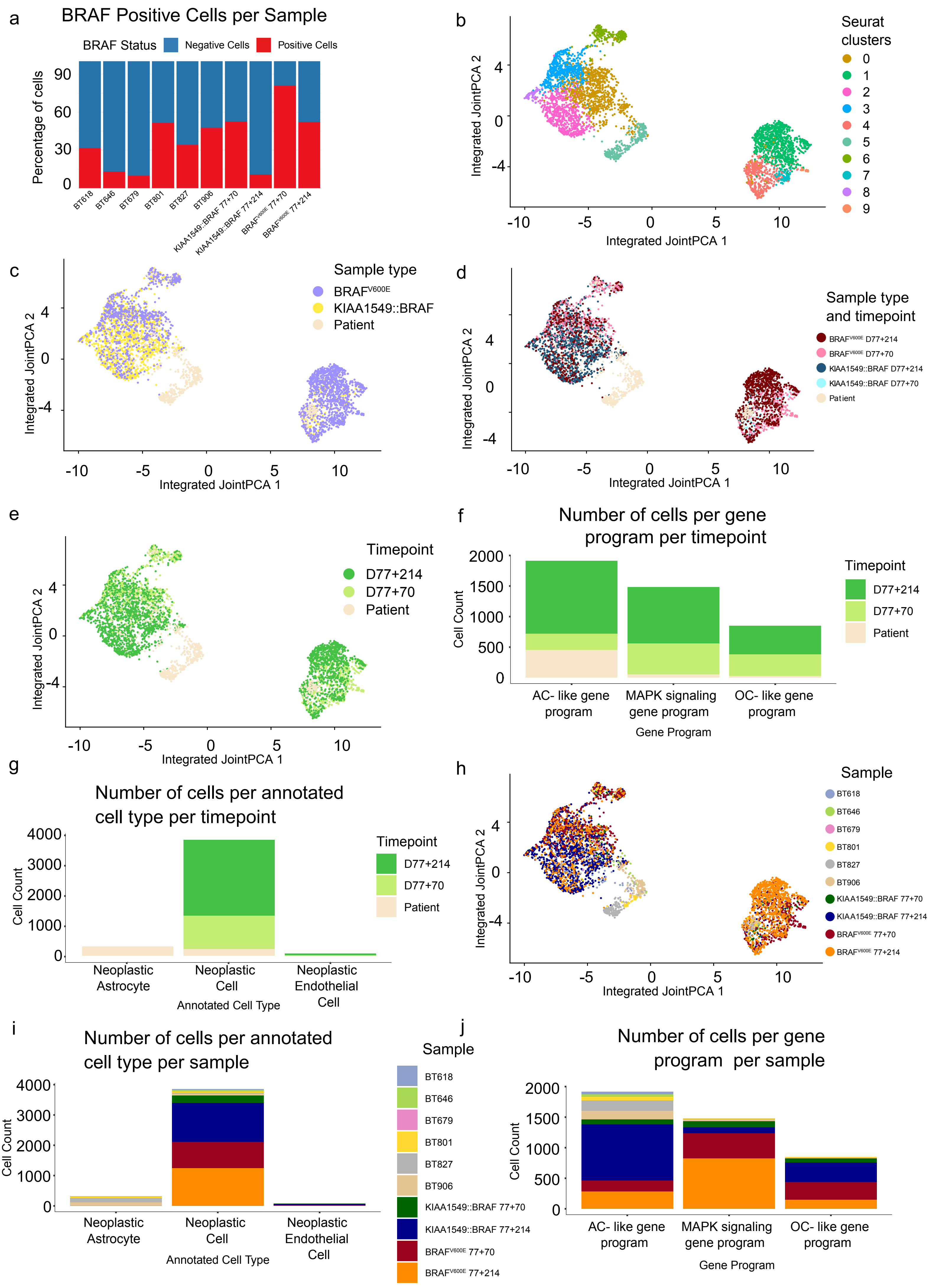

Supplementary Figure 6

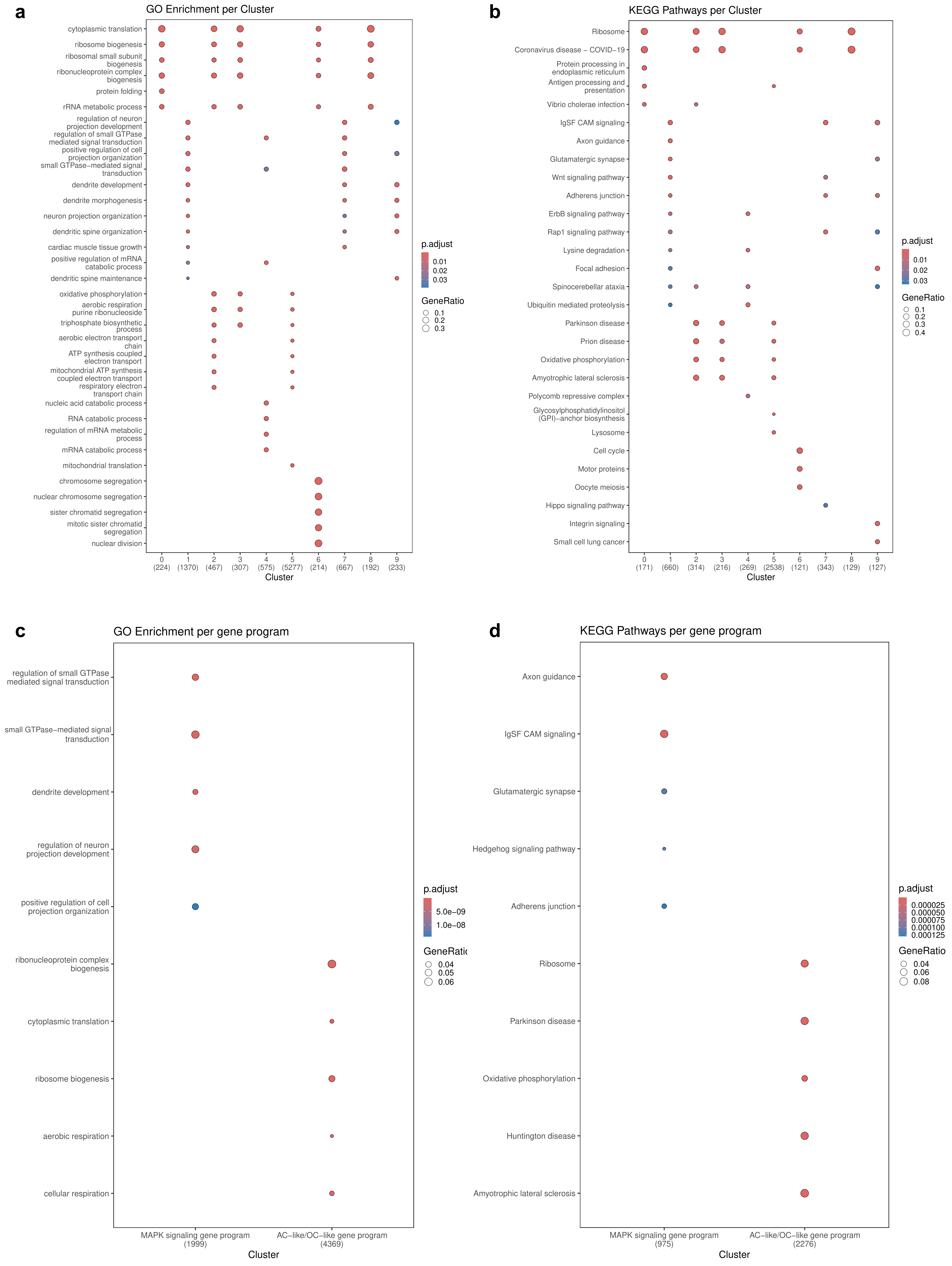

Supplementary Figure 7

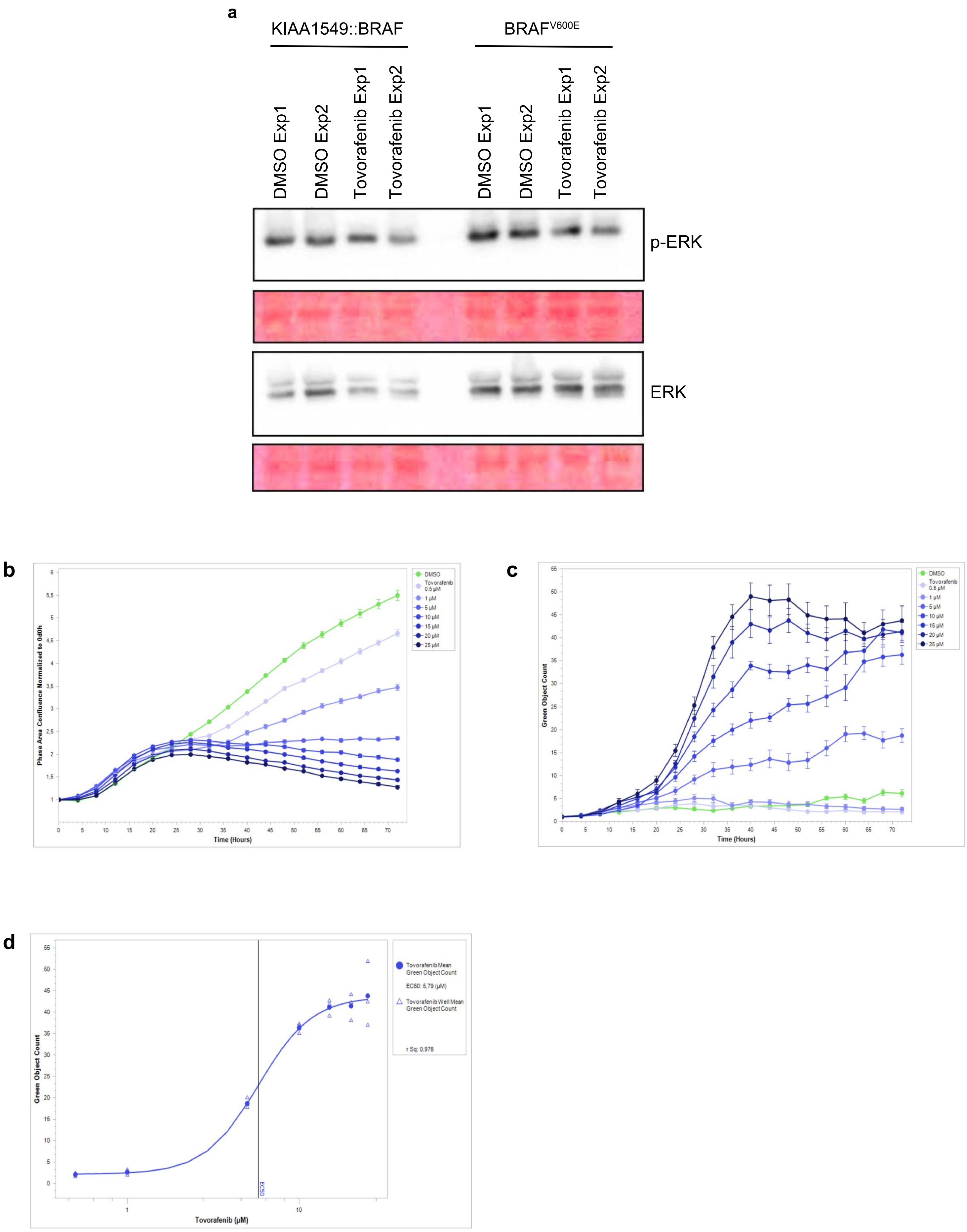

Supplementary Figure 8
